# A systematic literature review of indicators measuring food security

**DOI:** 10.1186/s40066-023-00415-7

**Published:** 2023-05-05

**Authors:** Ioannis Manikas, Beshir M. Ali, Balan Sundarakani

**Affiliations:** Faculty of Business, University of Wollongong in Dubai, Knowledge Park, 20183 Dubai, United Arab Emirates

**Keywords:** Data, Food insecurity, Index, Indicators, Measurement, Scale

## Abstract

Measurement is critical for assessing and monitoring food security. Yet, it is difficult to comprehend which food security dimensions, components, and levels the numerous available indicators reflect. We thus conducted a systematic literature review to analyse the scientific evidence on these indicators to comprehend the food security dimensions and components covered, intended purpose, level of analysis, data requirements, and recent developments and concepts applied in food security measurement. Data analysis of 78 articles shows that the household-level calorie adequacy indicator is the most frequently used (22%) as a sole measure of food security. The dietary diversity-based (44%) and experience-based (40%) indicators also find frequent use. The food utilisation (13%) and stability (18%) dimensions were seldom captured when measuring food security, and only three of the retrieved publications measured food security by considering all the four food security dimensions. The majority of the studies that applied calorie adequacy and dietary diversity-based indicators employed secondary data whereas most of the studies that applied experience-based indicators employed primary data, suggesting the convenience of collecting data for experience-based indicators than dietary-based indicators. We confirm that the estimation of complementary food security indicators consistently over time can help capture the different food security dimensions and components, and experience-based indicators are more suitable for rapid food security assessments. We suggest practitioners to integrate food consumption and anthropometry data in regular household living standard surveys for more comprehensive food security analysis. The results of this study can be used by food security stakeholders such as governments, practitioners and academics for briefs, teaching, as well as policy-related interventions and evaluations.

**Supplementary Information:**

The online version contains supplementary material available at 10.1186/s40066-023-00415-7.

## Introduction

Providing sufficient, affordable, nutritious, and safe food for the growing global population remains a challenge for human society; this task is made further difficult when governments are expected to provide food security without causing climate change, degrading water and land resources, and eroding biodiversity [1]. As long as food self-sufficiency and citizens’ wellbeing depend on sustainable food security, food security will remain a global priority [2, 3]. According to the 1996 World Food Summit definition, food security is achieved ‘when all people, at all times, have physical and economic access to sufficient, safe and nutritious food to meet their dietary needs and food preferences for an active and healthy life’ [4].

This definition by the Food and Agriculture Organization has laid the foundation for the four food security dimensions [5]: *availability*, *access*, *utilisation*, and *stability*. Relatedly, any kind of food security analysis, programme, and monitoring, with respect to predefined targets, requires valid and reliable food security measurement. However, measuring such a non-observable concept as a latent construct has remained challenging because of its complex and evolving nature: it has many dimensions and components [6], and involves a *continuum of situations*, invalidating the application of dichotomous/binary measures [7]. Food security measurement poses two fundamental yet distinct problems [8]: determining *what is being measured* and *how it is measured*. The *what* question refers to the use of appropriate indicators for the different dimensions (availability, access, utilisation, and stability) and components (quantity, quality, safety, and cultural acceptability/preference), while the *how* question refers to the methodology applied for computing the indicators (i.e. data, methods, and models).

Scholars have proposed a variety of indicators to measure food security. Over this time, the definition and operational concept of food security has changed as well, and, with it, the type of indicators and methodologies used to gauge it. One such important change is the paradigm shift ‘from the global and the national to the household and the individual, from a food-first perspective to a livelihood perspective, and from objective indicators to subjective perception’ [6]. Despite the call to harmonize measurements for better coordination and partnerships, to date, there remains no consensus among governments, quasi-legal agencies, or researchers on the indicators and methodologies that should be applied for measuring and monitoring food security at global, national, household, and individual levels [9]. Instead, an overabundance of indicators makes it difficult to ascertain which indicators reflect which dimensions (availability, access, utilization, or stability), components (quantity, quality, safety, cultural acceptability/preferences), and levels (global, national, regional, household or individual) of food security [10]. The number of food security dimensions or components assessed also greatly vary in the literature. Indicators that assess only a specific dimension or component oversimplify the outcomes and do not reveal the full extent of food insecurity, for example. Although such highly specific indicators do help conceptualise and reveal food insecurity, they still fail to accurately show trade-offs among the different dimensions, components, and intervention strategies. There is ultimately a possibility of shifting the food insecurity problem from one dimension/component to another.

The practical limitations of existing food security measurements were once again exposed by 2019 coronavirus pandemic (COVID-19), the Scientific Group for the United Nations Food Systems Summit [11] that ‘the world does not have a singular source of information to provide real-time assessments of people facing acute food insecurity with the geographic scale to cover any country of concern, the ability to update forecasts frequently and consistently in near real-time’. They further stated that current early warning systems lack suitable indicators to monitor the degradation of food systems. Aggravating this problem, these measurement indicators are not standardised, making comparisons among indicators over space and time complicated [9]. First, some of the indicators are composite indicators measuring two or more food security dimensions, whereas others measure individual dimensions. Second, some of the indicators focus on factors contributing to food security than on food security outcomes. Third, some indicators are quantitative, whereas others are qualitative measures based on individuals’ perceptions. Fourth, the levels of analysis greatly vary as well because some indicators are global and national measures, whereas others are household and individual measures. Fifth, the intended purposes of the indicators range from advocacy tools to monitoring and evaluating progress towards defined policy targets.

Although numerous food security indicators have been developed for use in research, there is no agreement on the single ‘best’ food security indicator among scientists or practitioners for measuring, analysing, and monitoring food security [12, 9]. The different international agencies also use their own sets of food security indicators (e.g. World Food Programme: Food Consumption Score (FCS), United States Agency for International Development (USAID): Household Food Insecurity Access Scale (HFIAS); FAO: Prevalence of Undernourishment (POU) and Food Insecurity Experience Scale (FIES); and Economic Intelligence Unit (EIU): Global Food Security Index (GFSI)). An ideal food security indicator should capture all the four food security dimensions at individual level (rather than at national or regional or household levels) to reflect the 1996 World Food Summit definition of food security. However, most of the available indicators are measures of food access at the household level.^1^ In practical use, only a few indicators that ‘satisfactorily capture each requisite dimension of food security and that are relatively easy to collect can be identified and adopted at little detriment to a broader agenda’ [9], which we attempt herein. In the light of the foregoing discussion, the main objective of this study was to critically review food security indicators and methodologies published in scientific articles using systematic literature review (SLR). The specific objectives were as follows:

1. To identify and characterize food security indicators with respect to dimensions and components covered, methods and models of measurement, level of analysis, data requirements and sources, intended purpose of application, and strengths and weaknesses;
2. To review and summarise the scientific articles published since the last decade by the indicators used, intended purpose, level of analysis, study region/country, and data source;
3. To quantitatively characterize the food security dimensions and components covered in the literature, and to review scientific articles that measured all the four food security dimensions; and
4. To identify and review recent developments and concepts applied in food security measurement.

Although there exist a few review studies on food security measurement in the literature (e.g. [8, 10, 13–15], the present study is more comprehensive as it covers a wide range of food security indicators, levels of measurement, and analysis of data requirements and sources. Moreover, unlike the existing review studies in the literature, the current study applies the SLR methodology to the analysis of food security indicators and measurement.

## Methods

### Review methodology

We followed a two-stage approach in this review. First, we identified the commonly used food security indicators based on recent (review) articles on food security measurement [8–10, 14, 15]. Using the retrieved information from these articles (and their references), the identified indicators were characterised (in terms of the dimensions and components covered, methods of measurement, level of analysis, intended uses, validity and reliability, and data requirements and sources). Tables 1, 2, 3, 4 present the summary of the characterisation of the identified food security indicators: experience-based indicators (Table 1), national-level indicators (Table 2), dietary intake, diversity and expenditure-based indicators (Table 3), and indicators reflecting coping strategies and anthropometry measures (Table 4). This first-stage analysis was used to address the first objective of the study. In the second stage, the SLR was conducted.

**Table 1:** Summary of characterization of experience-based food security indicators

Indicators	Description/method	Recall period	Level of analysis	Dimension covered	Components covered	Possible purpose	Validity and Reliability	Data requirement and availability	Strengths and weaknesses
Household Food Security Survey Module (HFSSM) (Bickel [111])	Measures households’ experience of FI using 18-item questionnaire (the 8 questions only for households with children) Used to monitor FS in USA Categorical scale based on the sum of affirmative responses (4 categories) -Continuous scale (0–9.3) based on a Rasch model with cut-off points	1 year; 1 month possible	Household	Access	Quantity, perceived quality	- Estimate FI prevalence - Monitor trends in FI at national level Program monitoring, evaluation and targeting	Valid and reliable [10], Gulliford et al. [17])	Can be collected using the 18-items questionnaire	-It is simple to implement, and has also a validated short version with 6-questions. It does not quantify/assess ‘actual’ food consumption, and diet quality
Food Insecurity Experience Scale (FIES) (FAO [18])	Measures individual’s experience of FI using an 8-item questionnaire Used by FAO to monitor global FS Categorical scale based on the sum of affirmative responses (4 categories) Continuous scale based on a Rasch model with cut-off points	1 year	Individual	Access	Quantity, perceived quality	Estimate prevalence of FI FS monitoring at global (SDG) and national levels Program monitoring and evaluation	Valid and reliable	Can be collected via the 8-items questionnaire For most countries data is available from FAO’s survey via Gallup Inc	Comparable FS estimates across countries/cultures/sub-populations; FAO provides software program and learning materials for computing FIES. Yet, FIES is complicated for non-specialists, does not quantify the ‘actual’ food consumption and diet quality, does not measure child FS
Household Food Insecurity Access Scale (HFIAS) (Coates et al. [112])	Measures households’ experience of FI using a 9-item questionnaire Categorical scale derived from the affirmative responses (4 categories) Continuous scale can also be derived based on a Rasch model	4 weeks	Household	Access	Quantity, perceived quality, Preference	Monitoring and evaluation of FS programs, Targeting Estimate FI prevalence	Valid and reliable [10]’ [20],	Can be collected via the 9-items questionnaire	It has a specific question about the ‘food preference’ component; straightforward to apply. Yet, it does not quantify the ‘actual’ food consumption and diet quality
Latin American and Caribbean Household Food Security Scale (ELCSA) [10], FAO [21])	Adapted from HFSSM to measure households’ experience of FI using a 15-item questionnaire (the 7 are questions only for households with children) Categorical scale based on the sum of affirmative responses (4 categories) Continuous scale based on a Rasch model with cut-off points	3 months	Household	Access	Quantity, perceived quality	Estimate FI prevalence Program monitoring, evaluation and targeting	Valid and reliable [10]	Can be collected via the 15-items questionnaire	Harmonised for its application in Latin American and Caribbean; easy to apply. A continuous scale based on a Rasch model can also be derived. ELCSA does not quantify the ‘actual’ food consumption and does not consider diet quality
Household Hunger Scale (HHS) (Deitchler et al. [22])	Estimates the prevalence of severe experiences of lack of food access and experiences of hunger HHS is derived from data collected using the last three HFIAS questions Categorical scale based on the sum of affirmative responses (3 categories)	30 days	Household	Access	Quantity	Estimate prevalence of severe FI or hunger across contexts Early warning for humanitarian responses	Valid and reliable [10]	Can be derived from HFIAS dataset or data collected via the 3-items questionnaire	Comparable across contexts; easy to apply. Yet, it does not quantify the ‘actual’ food consumption and does not consider diet quality
Food Adequacy Questionnaire (FAQ) [23]	Subjective indicator of FS based on individual’s self-reported adequacy of food	–	Household/Individual	Access	Quantity	Estimate prevalence of FI Rapid FS assessment	Not valid and reliable [24]	Data can easily collected using one question	Capture the behavioural aspects of FI, Suitable for conducting preliminary FS assessments Yet, prone to subjective biases

*FS* food security, *FI* food insecurity

**Table 2:** Summary of characterization of national level food security indicators

Indicators	Description/method	Recall period	Level of analysis	Dimension covered	Components covered	Possible purpose	Validity and Reliability	Data requirement and availability	Strengths and weaknesses
Prevalence of undernourishment (POU) [13],FAO [113])	Estimates the proportion of a population with dietary energy deficiency relative to the minimum calorie requirement of an average individual in the population Derived from three parameters: amount of available calorie for domestic consumption, minimum amount of calorie required by the population, and inequality in access to the available calories Expressed as a percentage of undernourished households relative to the total population	1 year	National	Availability	Quantity	Estimate prevalence of undernourishment Monitor trends in undernourishment at national and global levels	Not valid, and low reliability [8, 13]	National food balance sheets (FAO); Household Income and Expenditure Surveys; official data on population size and composition	It provides insight into levels and trends of undernourishment; facilitate global and regional FS governance. Yet, it does not provide information on the actual distribution of the number of hungry people within the population; proxy of calorie availability for calorie intake; energy deficiency is not a valid measure of FI; micronutrients are not considered, overstate food availability in FBS due to not accounting for food losses
Global Food Security Index (GFSI) [26]	Composite index (0–100) by aggregating multiple indicators, using expert weights, or weighting methods: DEA and PCA	1 year	National	All four	Quantity, Quality, Safety	Analysing the factors influencing food security Monitoring FS at global level	Valid and reliable	EIU	It provides insights into the vulnerability of a nation’s food system by attributing to the causes. Yet, it focuses on analysis of FS determinants, and do not measure FS outcomes
Global Hunger Index (GHI) (Pangaribowo [27], Wiesmann [28])	Composite indicator for analysing the extent, trend and cause of hunger worldwide Calculated as the arithmetic mean of three components of hunger: undernourishment, child underweight and stunning and child mortality Five GHI categories, which are determined based on randomly selected cut-off points	1 year	National	Access, Utilisation	Quantity, Quality, Safety	Estimate prevalence of hunger Monitor trends in poverty at national and global levels	Not valid and low reliability as it depends on POU	FAO (prevalence of undernutrition), WHO (child underweight), UNICEF (child morality)	It provides insight into levels & trends of hunger; facilitate global and regional poverty governance. Suffers from double counting arising from the correlation of the three components of hunger used to construct GHI
Suite of Food Security index [29, 30]	Composite index (0–100) covering all the four dimensions of FS. The multiple dimensions and indicators are normalised, and then aggregated using a set of weights, for example based on PCA	1 year	National	All four	All	Estimate prevalence of FI Monitor trends in FS at national and global levels	Validity and reliability not demonstrated	FAO compiles annual data for different countries, and are openly accessible at FAOSTAT	It accounts for all the FS dimensions and components, provides insight into levels & trends of FI; facilitate global and regional FS governance, availability of data from FAO. No standardised weighting and normalisation methods

FS food security, *FI* food insecurity, *EIU* economic intelligence unit

**Table 3:** Summary of characterization of dietary intake, diversity and expenditure-based food security indicators

Indicators	Description/method	Recall period	Level of analysis	Dimension covered	Components covered	Possible purpose	Validity and reliability	Data requirement and availability	Strengths and weaknesses
Food consumption score (FCS) (WFP [31])	Composite indicator that considers dietary diversity, consumption frequency, and diet quality (nutritional values of various food groups) Given $f$ as the number of days each food group was consumed within the last seven days, FCS is measured as: $FCS=2{f}_{staple}+3{f}_{pulse}+1{f}_{vegetable}+1{f}_{fruit}+4{f}_{animal, fish}+4{f}_{dairy}+0.5{f}_{sugar}+0.5{f}_{oil}$ The total score (0–112) is used to classify households into one of the three groups based on the thresholds	7 days	Household	Access	Quantity, Quality	- Estimate FI prevalence - Program monitoring, evaluation and targeting	Not valid and reliable [32]	Dietary records, Food Frequency Questionnaires Household Consumption and Expenditure Survey	Easy to apply and allows for rapid FS assessment. Yet, it does not account for amount/quantity of food consumed, micronutrient intakes and intra-household allocations, the cut-off points (thresholds) for classifying households into different classes are not valid; Prone to measurement error as it relies on respondent memory
Household dietary diversity score (HDDS) (Swindale and Bilinsky [33])	A qualitative measure by counting the number of food groups (of 12 groups) consumed by a household within the recall period: cereals; roots/tubers; vegetables; fruits; meat/poultry/offal; eggs; fish/seafood; pulses/legumes/nuts; milk/milk products; oil/fats; sugar/honey; and miscellaneous (others). The FAO version contains 16 food groups	1 or 7 days	Household	Access	Quantity, Quality	- Estimate FI prevalence - Program monitoring, evaluation	Not valid and reliable [32]	Dietary records, Food Frequency Questionnaires Household Consumption and Expenditure Survey	Simple and easy to understand It has not been validated as a proxy for adequacy of particular macronutrients or micronutrients (diet quality). There is no universally accepted cut-off for this indicator Prone to measurement error as it relies on respondent memory
Women’s and individual dietary diversity scores [34]	A qualitative measure of individual’s access to a diverse of food items. It reflects dietary quality by accounting the adequacy of micronutrient of the diet) A simple count of food items consumed of the 16 food groups: cereals; vitamin A–rich vegetables and tubers; white roots and tubers; dark-green leafy vegetables; other vegetables; vitamin A–rich fruits; other fruits; organ meat; flesh meat; eggs; fish; legumes, nuts, and seeds; dairy; oils and fats; sweets; condiments)	1 or 7 days	Individual	Access	Quantity, Quality	- Estimate FI prevalence at individual level - Program monitoring, evaluation and targeting	Validated for women aged 15–49 years old, and reliability not checked [10]	Dietary records, food frequency questionnaires	Provides insights into adequacy of macro- and micronutrients at individual level. Also, proven to be a good measure of household macronutrient adequacy and household nutrition insecurity. Prone to measurement error as it relies on respondent memory
Calorie Adequacy Indicator [35, 36]	Measures a household’s calorie adequacy (the availability of calorie relative to the calorie requirement of the household by accounting for age and sex differences OR the amount of expenditure on food that is required to purchase the minimum caloric requirement -two categories: adequate calorie, food secure, and inadequate calorie food insecure	1 year/1 week/1 month	Household	Access	Quantity	-Estimate FI prevalence - program monitoring, evaluation and targeting	Like POU, not a valid measure of FS, and low reliability	Dietary records, Food Frequency Questionnaires Household Consumption and Expenditure Survey	It provides insight into levels & trends of household level undernourishment. Yet, it does not account for dietary quality as energy deficiency is not a valid measure of FI; micronutrients are not considered; does not account for inequality in access to the available calories

*FS* food security, *FI* food insecurity

**Table 4:** Summary of characterization of food security indicators reflecting Coping Strategies and Anthropometry Measures

Indicators	Description/method	Recall period	Level of analysis	Dimension covered	Components covered	Possible purpose	Validity and Reliability	Data requirement and availability	Strengths and weaknesses
Coping Strategy Index (CSI) (Maxwell and Caldwell [37])	Assess what people do when they cannot access enough food A total of 12 to 15 coping strategies are identified through focus groups, and these strategies are assigned into one of the four categories: Dietary change, Short-term measures to increase food availability, Short-term measures to decrease the number of people to feed and Rationing Calculated as a weighted average of the frequency of the coping strategies as: ${CSI}_{i}=\sum {w}_{j}{S}_{ij}$, where ${w}_{j}$ is the weight assigned to *j*^*th*^ coping strategy (${S}_{ij}$) used by the *i*^*th*^ household. The weight (${w}_{j}$) ranges between 1 (least severe category) to 4 (most severe coping behaviour)	7 day	Household	Access	All	Analysis of causes and consequences of FI, Impact evaluation, Monitoring Early warning	Valid, but reliability not checked [10]	Data collection on a series of questions on how households are responding to food shortages, through focus group discussions	Relatively simple and allows rapid assessment. Yet, it is a context-specific measure, and relatively expensive. Also, no standardised cut-off points
Reduced CSI	Same with CSI, but allows FS comparison across different contexts by using five predefined coping strategies, with universal set of severity weightings: Eating less-preferred foods (1.0), Borrowing food/money from friends and relatives (2.0), Limiting portions at mealtime (1.0), Limiting adult intake (3.0), and Reducing the number of meals per day (1.0). The weighted sum of the frequencies of these five strategies (0–56) used for assigning households	7 day	Household	Access	Quantity, Quality, Preference	Surveillance, in the early stages of FS crises, and can also be combined with the HHS for analysing serious and prolonged FS crises	Valid, but reliability not checked [10]	Data collection on the five predefined strategies on how households are responding to food shortages, through focus group discussions	Standardised for different contexts, relatively simple and allows rapid assessment. Yet, relatively expensive, and no agreed threshold/cut-off for interpreting the scores
Anthropometry measures	-Assessing individuals’ nutritional outcomes in relation to the height, weight and body size of individuals - Simple calculations, and Z-score with cut-off points	–	Individual	Utilisation	Quantity, quality, safety	-Measuring the effect of undernutrition on individuals’ health and wellbeing - Mapping of nutritional security -Evaluating relief and emergency programs	Reliable and valid	WHO; Demographic and Health Surveys; Multiple Indicator Cluster Surveys	Highly standardized, allow mapping of nutritional security at national and local levels. Yet, they are indirect measure of FS, generally expensive and time consuming [38]

*FS* food security, *FI* food insecurity uncategorized references

### Literature searching and screening processes

We applied the SLR methodology to systematically search, filter, and analyse scientific articles on food security measurement. The SLR is a commonly applied and accepted research methodology in the literature [39]. Although the SLR methodology is widely applied in different disciplines such as the health and life sciences, its application in economics is limited. However, it has recently been applied in agricultural economics (e.g. [40–43]. In this study, we closely followed the six steps of a systematic review process [39], namely, (a) defining research questions, (b) formulating search strings, (c) filtering studies based on inclusion and exclusion criteria, (d) conducting quality assessment of the filtered studies, (e) collecting data from the studies that passed quality assessment, and (f) analysing the data. The literature screening process that we followed is also in line with the guidelines in the Preferred Reporting Items for Systematic Reviews and Meta-Analyses Statement (PRISMA) [44].

**Fig. 1:**
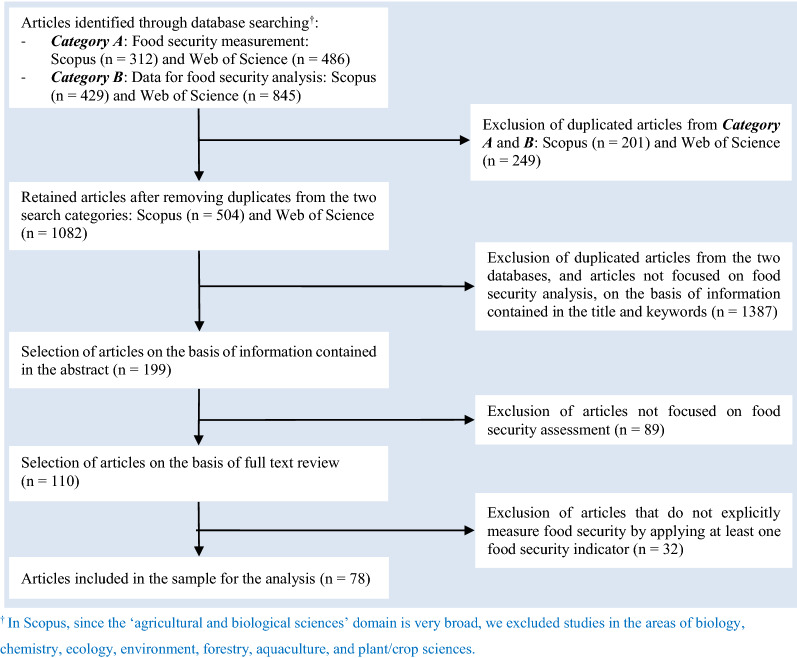
Literature searching and screening criteria

The bibliographic databases of Scopus and Web of Science (WoS) were used to search scientific articles on food security measurement (i.e. indicators, data, and methods) and help us answer the research question ‘How has food in/security been measured in the literature?’ Two categories of search strings were applied: One focussing on food security indicators (*Category A*), and another one on data requirement and sources of food security measurement (*Category B*). Specifically, the search strings (“food security” OR “food insecurity” OR “food availability” OR “food affordability” OR “food access” OR “food utilization” OR “food utilisation” OR “food stability” OR “nutrition security” OR “nutrition insecurity”) AND (“measurement” OR “indicators” OR “metrics” OR “index” OR “assessment” OR “scales”) were used for *Category A*. For *Category B*, we used (“food security” OR “food insecurity” OR “food availability” OR “food affordability” OR “food access” OR “food utilization” OR “food utilisation” OR “food stability” OR “nutrition security” OR “nutrition insecurity”) AND (“data” OR “big data” OR “datasets” OR “survey” OR “questionnaire”). The retrieved articles together with some of the inclusion and exclusion criteria, and the number of retrieved articles at each step, are presented in Fig. 1. The following inclusion and exclusion criteria were also used during the literature searching and screening process in addition to those criteria presented in Fig. 1: (a) Search field: title–abstract–keywords (Scopus); topic (WoS), (b) Time frame: 2010–09/03/2021, (c) Language: English, (d) Field of research: Agricultural and Biological Sciences^2^; Economics, Econometrics and Finance (Scopus); Agricultural Economics Policy; Food Sciences Technology (WoS), and (e) Type: journal articles (*Category A*); journal articles, data, survey, database (*Category B*). We limited our literature search to publications from 2010 onwards since it was during this period that due attention has been given to the harmonisation of food security measurement.^3^ This was also evident from the 2013 special issue of *Global Food Security* journal on the theme *Measuring Food and Nutrition Security*.^4^

As we noted above, an ideal food security indicator should capture all the four food security dimensions at individual level to reflect the 1996 World Food Summit definition of food security. We reviewed only those articles that have explicitly measured food in/security by applying at least one food security indicator. These indicators, measuring at least one of the four food security dimensions, were identified based on recent (review) articles on food security measurement [8–10, 14, 15]. A total of 110 articles were selected for full content review after the pre-screening process based on title, keyword and abstract review (Fig. 1). After the full content review, 32 articles were further excluded. Fourteen of these were excluded, as they did not measure food security explicitly (e.g. [45, 46] or the food security indicator/method of measurement was not described (e.g. [47] or they used ‘inappropriate’ indicators that do not capture at least one of the four food security dimensions (e.g. [48]. For example, Koren and Bagozzi [48] used per capita cropland as a food security measure, which is not a valid indicator for the multidimensional food security concept (it cannot even fully capture the food availability dimension). Thirteen publications that we classified as methodological, two review articles [49, 50], and three articles on seed insecurity [51], marine food insecurity [52] and political economy of food security [53] were also excluded. Finally, we reviewed, analysed, and summarised the scientific evidence of 78 articles on food security measurement (see Additional file 1 for the list of the articles and the data). The validity and reliability of the SLR have been ensured by specifying the SLR setting following Kitchenham et al. [39], and by providing sufficient information regarding the literature extraction and screening processes. Moreover, the three authors have double-checked the correctness of the processes such as definitions of search strings and inclusion–exclusion criteria, and confirming the retrieved data and data interpretation to reduce bias. The limitations of the study are also discussed (see under the “Discussion” section).

## Results

### Review of articles by region, indicators used, intended purpose, and level of analysis

**Fig. 2:**
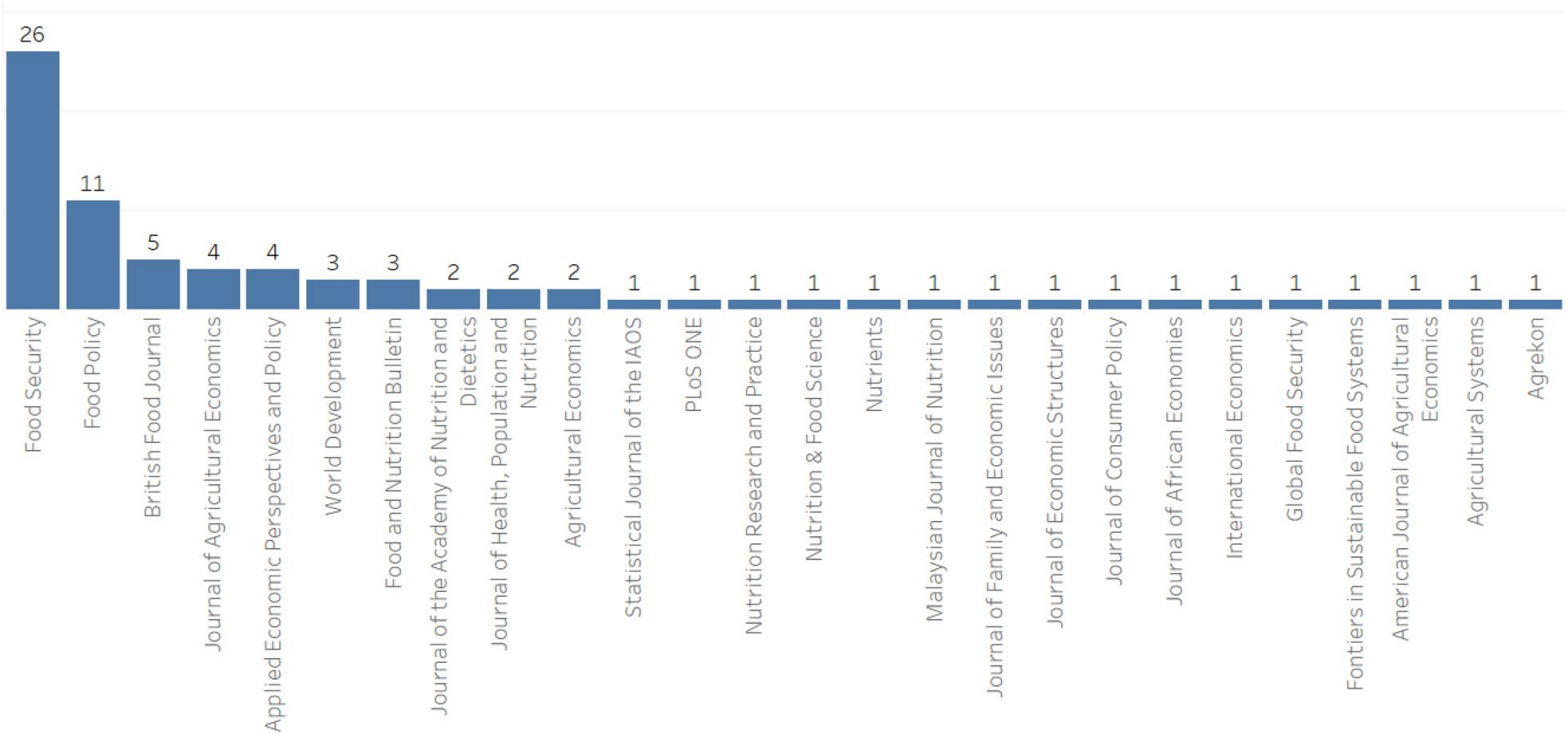
Number of articles per journal (total number of articles: 78)

**Fig. 3:**
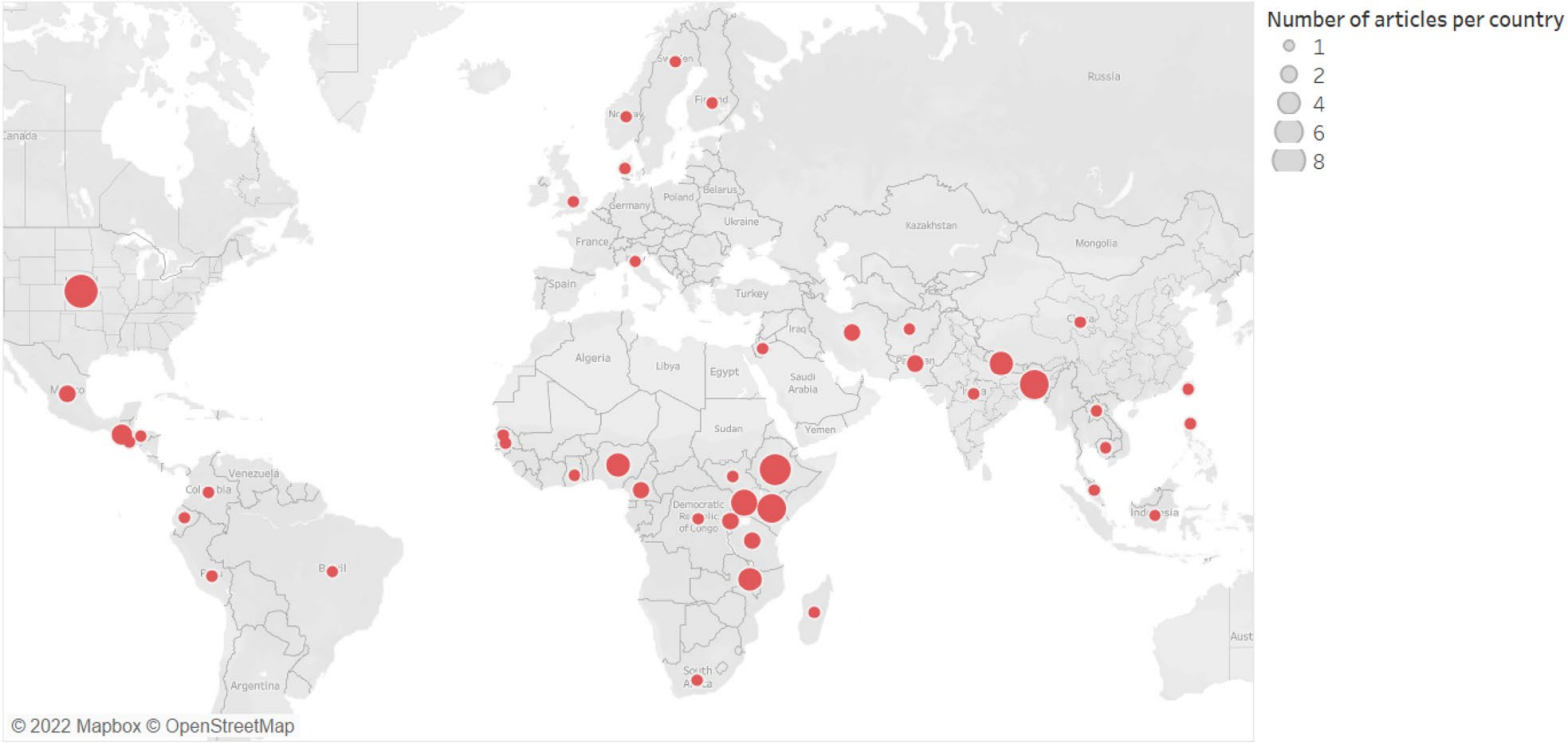
Summary of articles by country (Note: Some articles focus on more than one country, resulting in 89 articles by study area)

Following the exclusion of the non-pertinent articles (Fig. 1), 78 articles were included in our food security measurement dataset for the analysis (Additional file 1). Relatively, more publications were retrieved from the years 2019 and 2020 whereas there were no articles from 2010.^5^ The journals of *Food Security* (33%) and *Food Policy* (14%) are the main sources of the retrieved articles (Fig. 2). The journals in the field of agricultural economics are also important sources of the retrieved articles (15%). Figure 3 depicts the distribution of the retrieved articles by region/country of study focus. Sub-Sahara Africa has been the main focus of the studies, followed by Asia. At country level, USA (8 studies) and Ethiopia (7 studies) were the most studied countries. Besides the studies represented in Fig. 3, we identified nine other studies focusing at global and regional levels: global [7, 12, 54, 55], developing countries (Slimane et al. [56]), Middle East and North Africa (MENA) region [57], Latin America and Caribbean [58], and Sub Sahara Africa [59, 23]. Despite food insecurity being a global issue, there is lack of studies covering the different parts of the world (e.g. MENA region, Latin America and Europe).

**Fig. 4:**
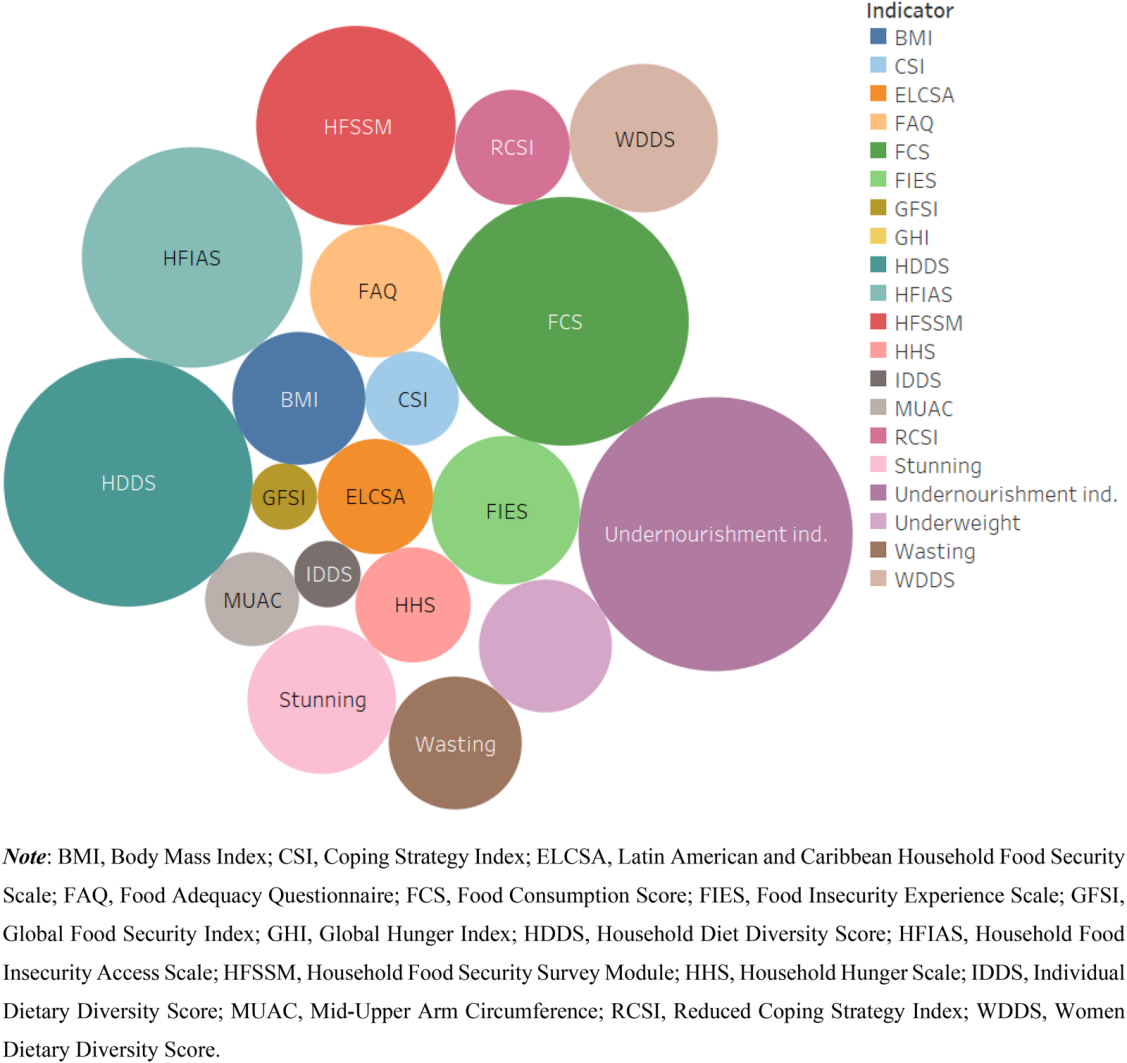
Summary of the publications by the type of food security indicators employed

Figure 4 shows the summary of the number of articles by the type of food security indicator that they applied. Seventeen articles applied the household-level calorie adequacy (i.e. undernourishment) indicator, making it the most frequently used one. This indicator measures calorie availability relative to the calorie requirement of the household by accounting for age and sex differences of the household members (note that this indicator is different from FAO’s Prevalence of Undernourishment (POU) indicator (Table 2; [13]). A household is considered as food insecure if the available calorie is lower than the household’s calorie requirement. This indicator has been used in the literature to assess the prevalence of food insecurity [35, 36, 60–67], for programme evaluation [68, 66], and to analyse food security determinants [35, 60, 66, 67, 69–71]. Some studies addressed the main drawback of the calorie adequacy indicator (its failure to account for diet quality) by measuring both calorie and micronutrient adequacy [54, 65, 70, 72].

Out of the 17 studies that applied the calorie adequacy indicator, three articles [35, 69, 71] classified households into food secure and food insecure based on the amount of expenditure on food that is required to purchase the minimum caloric requirement. A household is classified as food insecure if the expenditure on food is less than the predetermined threshold amount required for achieving the minimum caloric requirement. This measure allows us to account for the effect of food price inflation on household’s food access.

A subjective (self-reported) version of the household calorie adequacy indicator, the Food Adequacy Questionnaire (FAQ), was also used in 4 of the 78 articles (Fig. 4). Tambo et al. [73] and Smith and Frankenberger [74] measured food insecurity as the number of months of inadequate food provisioning during the last year owing to lack of resources. Bakhtsiyarava et al. [75] used FAQ to derive a binary measure of food security based on self-reported shortage of food in the last year, whereas Verpoorten et al. [23] measured food security using the question ‘Over the past year, how often, if ever, have you or anyone in your family gone without enough food to eat? Never/Just once or twice/Several times/Many times/Always’. Although these simple food security measures based on FAQ can usefully capture a household’s experience of food insecurity and for conducting preliminary assessments, they are prone to subjective biases [24]. A comparison of studies is complicated because FAQ’s measures are not standardised (e.g. differences in phrases and scales used in the questions).

The dietary diversity indicators Household Diet Diversity Score (HDDS), Women Diet Diversity Score (WDDS), Individual Diet Diversity Score (IDDS), and Food Consumption Score (FCS) were also frequently used in the literature (Fig. 4). About 44% of the publications used diet diversity indicators for measuring food security. (Additional file 2: Tables S1, S2) summarise the studies that applied the dietary diversity score measures (HDDS, WDDS, IDDS) and FCS. Most of the studies applied the diversity score indicators for estimating food insecurity prevalence (Additional file 2: Table S1). Bakhtsiyarava et al. [75], Bolarinwa et al. [76], Islam et al. [77], and Sibhatu and Qaim [78] applied HDDS when analysing the determinants of food security. Tambo et al. [73] and Islam et al. [68] used HDDS as a measure of food security for program evaluation.

The main weakness of the dietary diversity measures is that they do not account for the quantity and quality of the consumed diet (nutritional value); for instance, consumption of very small quantities of certain foods would raise the diversity score without contributing much to a household’s/individual’s nutritional and micronutrient supply [78]. HDDS does not also account for intra-household diet diversity. Thus, a higher diet diversity score does not necessarily mean a better household/individual food security. Most of the retrieved articles addressed these drawbacks by combining diversity measures with other food security indicators (Additional file 2: Table S1). For example, Sibhatu and Qaim [78] applied HDDS and WDDS in combination with measures of calorie and micronutrient adequacy. Tambo et al. [73] combined HDDS and WDDS with the Food Insecurity Experience Scale (FIES) and FAQ, whereas Bolarinwa et al. [76] integrated HDDS and per capita food expenditure.

There is also a difference in the literature regarding the recall period used when measuring dietary diversity, namely, 7 days vs 24 h (Additional file 2: Table S1). A 7 day recall period leads to higher diversity scores than a 24 h recall period because it considers the daily variation in food consumption [78]. Although the 7 day recall period is associated with higher respondent bias, conclusions drawn from a 24 h recall period may also be misleading, as some relevant food groups might not be considered in the food security assessment (e.g. livestock products that food insecure households seldom consume daily) [78]. It is therefore important to consider the differences in recall periods when designing measurement.

About 57% of the studies that employed FCS (Additional file 2: Table S2) used it to estimate food insecurity prevalence [36, 65, 70, 79–81, 83, 84]. Four other studies applied FCS to analyse the determinants of food security [85–88], whereas two used it for impact evaluation [89, 90].

D'Souza and Jolliffe [85] showed how applying two different food security indicators (per capita daily caloric intake and FCS) could lead to different conclusions when analysing the effect of food price shock on household food security. They estimated the marginal effects of wheat price increase on per capita daily caloric intake and FCS using unconditional quantile regression for each decile of the food security distribution. They found that households with lower calorie intake (food insecure households) did not exhibit a decline in per capita calorie intake because of the wheat price increase. However, households with higher calorie intake (food secure households) exhibited a higher reduction in per capita calorie intake in response to the price increase. On the other hand, the FCS estimation results showed that the most vulnerable households exhibited larger reductions in dietary diversity (FCS) in response to higher wheat prices compared with the households at the top of the FCS distribution (households with higher FCS). Thus, the most vulnerable households might maintain their calorie intake by compromising diet quality. These results imply that food security monitoring or impact assessments based solely on calorie intake could be misleading, and may have severe long-term implications for households’ well-being. In this regard, analysis based on dietary diversity-based measures (e.g. FCS) provides more insights into the effects of shocks on household food security (diet quality) across the entire food security distribution [85]. However, Ibok et al. [36] noted that FCS (and per capita calorie adequacy) are not good indicators of household’s vulnerability to food insecurity compared with CSI. In response, they developed the Vulnerability to Food Insecurity Index.

About 40% of the retrieved publications used experience-based indicators (Household Food Insecurity Access Scale [HFIAS], Household Hunger Scale [HHS], Household Food Security Survey Module [HFSSM], Latin American and Caribbean Household Food Security Scale [ELCSA], Food Insecurity Experience Scale [FIES]) for measuring food security (Fig. 4). HFIAS is the most widely used experience-based indicator (11 articles), followed by HFSSM (9 articles) and FIES (5 times). ELCSA and HHS have been used three times each. HFIAS was primarily used for estimating the prevalence of food insecurity, whereas its adapted version HHS was mainly used for analysing the determinants of food insecurity (Additional file 2: Table S3). The HFSSM was mainly used to analyse the determinants of household level food security in the US (six articles) (Additional file 2: Table S4). Courtemanche et al. [91] and Burke et al. [19] used HFSSM for program evaluation, respectively, to analyse the effects of Walmart Supercenters (which increase food availability at lower food prices) on household food security and school-based nutrition assistance programs on child food security (Additional file 2: Table S4).

Romo-Aviles and Ortiz-Hernández [92] used the ELCSA food security indicator to analyse the differences in food, energy, and nutrients supplies among Mexican households according to their food insecurity status (Additional file 2: Table S4). In the first stage, they applied an ordinal regression model to analyse the determinants of household food insecurity status. In the second stage, they analysed the effect of food insecurity (i.e. a household’s food insecurity state as an independent variable) on household’s energy and nutrient supplies by using the ordinary least squares (OLS) model. Sandoval et al. [66] compared ELCSA and the household calorie adequacy indicator in food security analysis: prevalence estimation, determinants analysis, and program evaluation. They concluded that the two indicators provided very different food insecurity prevalence estimates, and the determinants were shown to vary significantly. The results of the programme evaluation also showed that the magnitude of the effect of a cash transfer program was significantly larger when using the ‘objective’ undernourishment indicator than the ‘subjective’ ELCSA food security indicator.

The majority of the five studies that used the FAO’s FIES indicator analysed the determinants of food security at regional and global levels, whereas one study [73] used it for program evaluation to assess the effect of provisions of a plant health service on food insecurity prevalence among farming households (Additional file 2: Table S5).

**Fig. 5:**
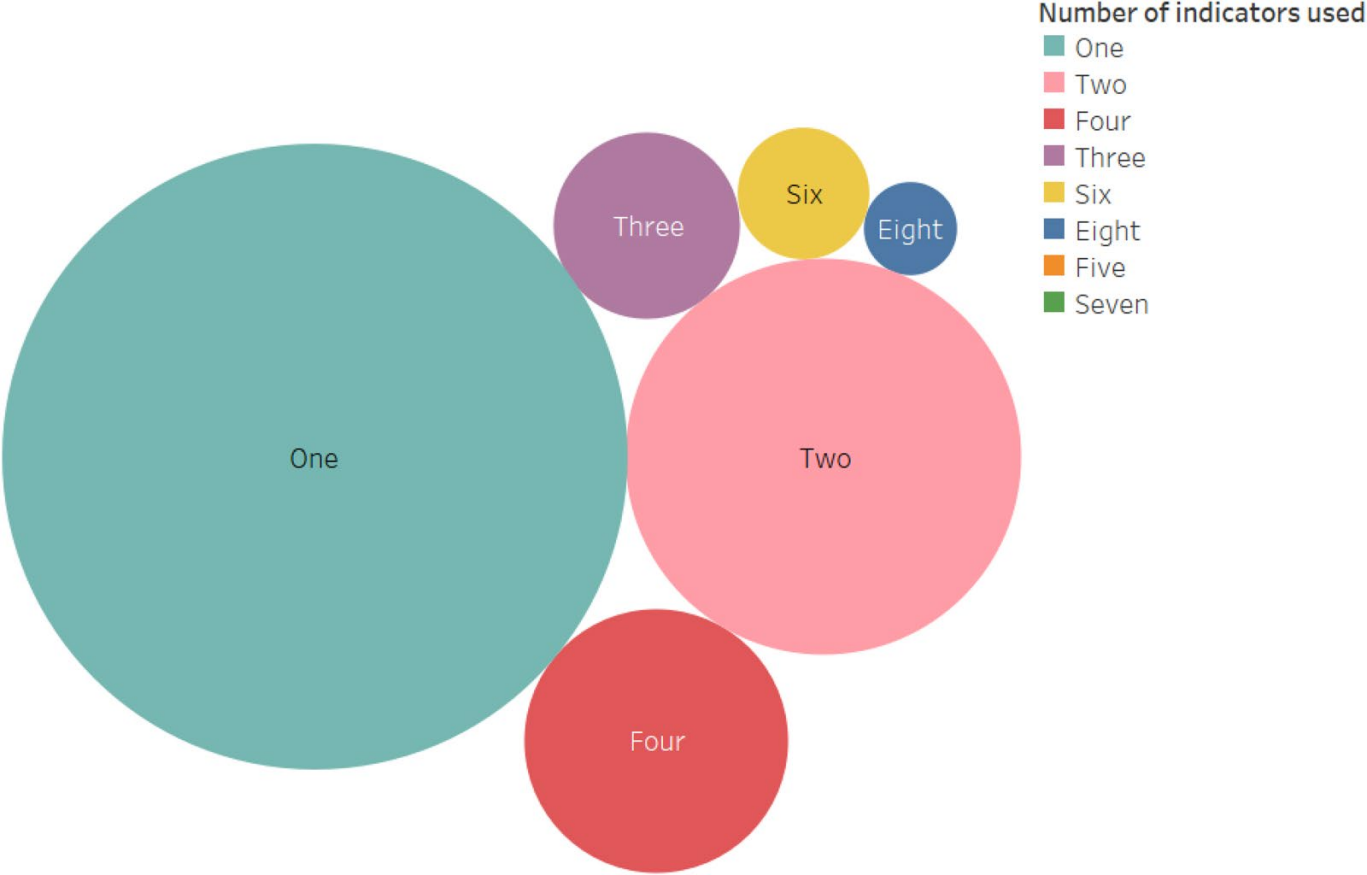
Summary articles by the number of indicators used per article (*N* = *78*)

Figure 5 summarises the data on the proportion of articles according to the number of indicators used per article. About 58% of the 78 articles used only one indicator in their food security analysis. The HFSSM and household calorie adequacy indicator have respectively been used eight and seven times as the sole food security indicator in food security analyses. HFIAS (four times), FIES (three times), and FCS (three times) were also used as the only measures of food security. The experience-based indicators (HFSSM, HFIAS, and FIES) are the most frequently used indicators as a single measure of food security in the literature, whereas the other categories of food security indicators (dietary diversity, anthropometric, and coping strategy) are mostly used in combination with other indicators.

Three studies (out of the 78 articles) applied at least six food security indicators (one study used eight indicators while the other two studies used six indicators each). Islam et al. [68] applied eight food security indicators to analyse the effects of microcredit programme participation on household food security. They applied the calorie adequacy indicator, HDDS (number of food groups consumed), Food Variety Score (FVS, number of food items consumed), three child anthropometry measures (stunning, wasting, underweight), and two women anthropometry measures (body mass index [BMI] and mid-upper arm circumference [MUAC]) as measures of food security. Bühler et al. [79] applied six indicators (FCS, Reduced Coping Strategy Index [RCSI], HFIAS, and child stunning, wasting and underweight) to evaluate the relationship between household’s food security status and individual’s nutritional outcomes. The indicators FCS, RCSI, and HFIAS were used to measure a household’s food security status, whereas the anthropometry measures were used as indicators of individual’s nutritional outcomes. Maxwell et al. [83] also applied six food security indicators (Coping Strategy Index [CSI], RCSI, FCS, HDDS, HFIAS, and HHS) to compare the estimates of food insecurity prevalence over seasons of the most frequently used indicators.

About 45% and 37% of the retrieved articles applied food security indicators to analyse food security determinants and for food insecurity prevalence estimation, respectively. The calorie adequacy indicator (11 articles), FCS (8 articles), HDDS (7 articles), HFSSM (7 articles), and HFIAS (7 articles) were the most frequently used indicators in this regard. The calorie adequacy indicator (11 articles), FCS (10 articles), HDDS (8 articles), and HFIAS (7 articles) were the most applied indicators for estimating food insecurity prevalence.

About 60% of the retrieved studies measured food security at household-level while 20% of them assessed food security at individual-level. The most frequently used household-level indicators were the calorie adequacy indicator (14 articles), FCS (13 articles), and HDDS (12 articles). The experience-based household food security indicators HFIAS and HFSSM were also used nine and seven times, respectively. For individual-level analyses, the following child anthropometry measures were mostly used: stunning (four times), wasting (three times), and underweight (three times). The individual-level food security indicators WDDS and BMI were also used four times each.

### Summary of indicators by study region and data source

**Fig. 6:**
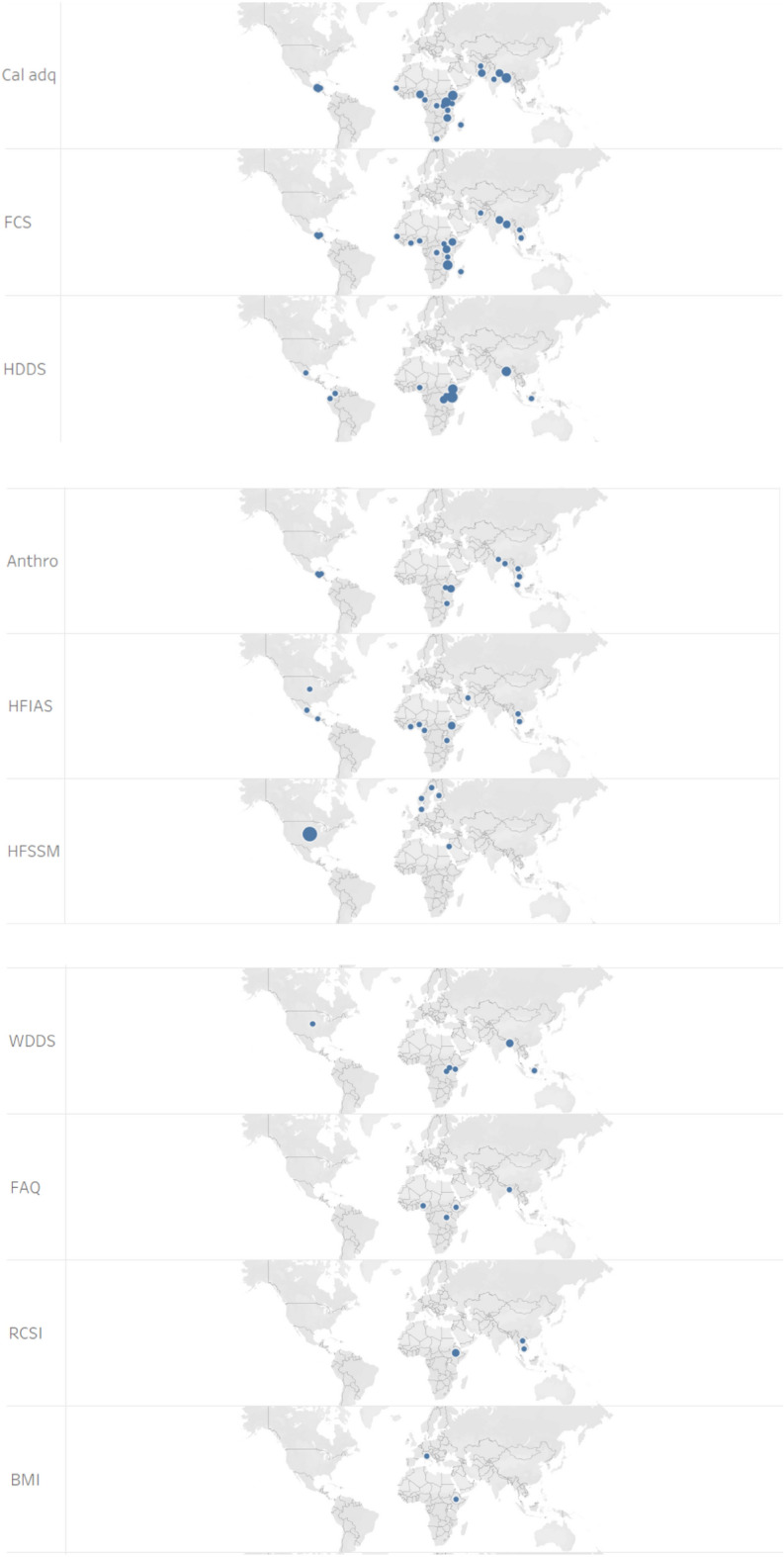

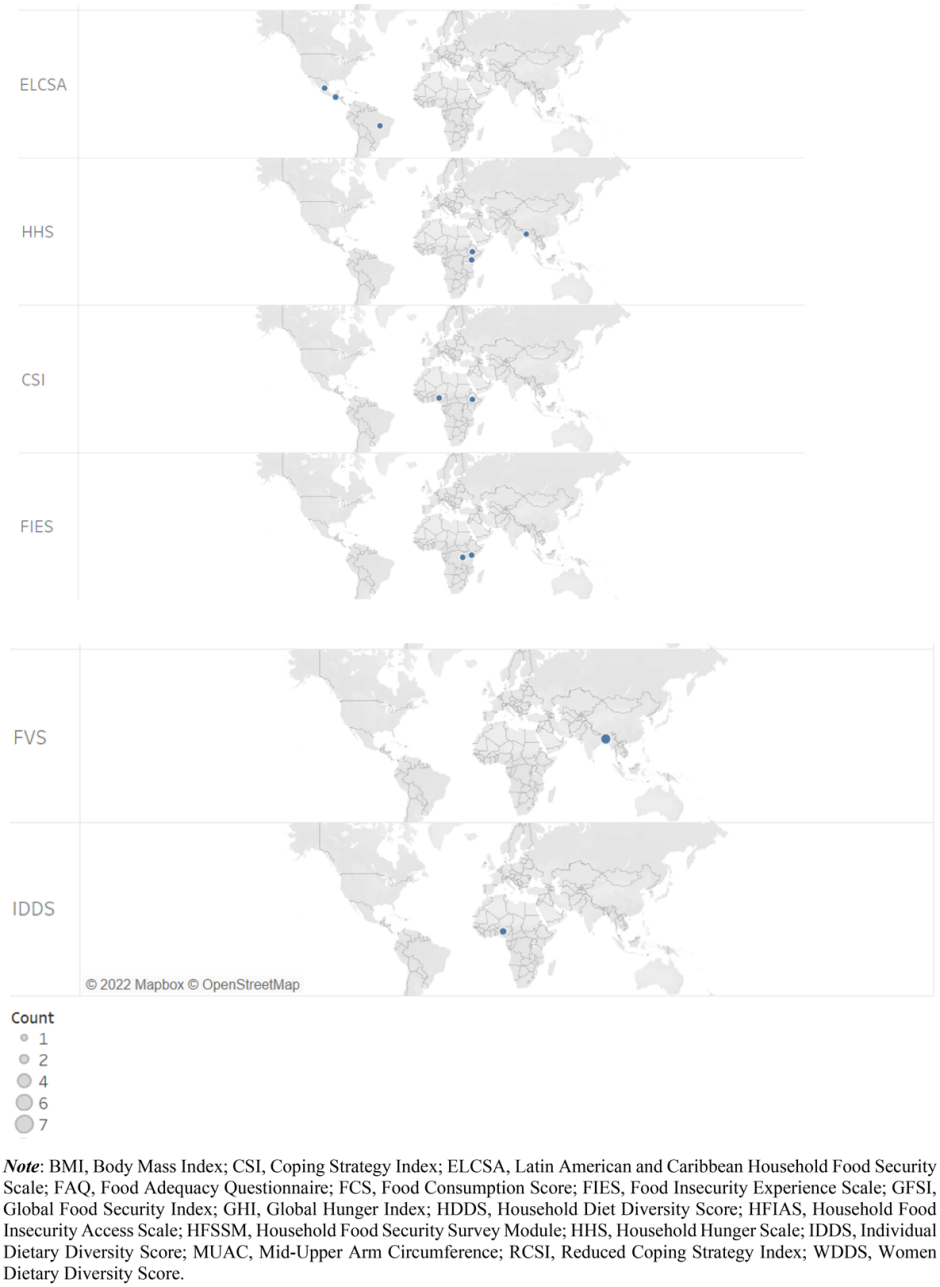
Summary of studies by country and indicators applied [Note: Multiple indicators could be used per study, and a study may cover multiple countries]

As shown in Fig. 3, the main focus areas of the 78 publications were Sub Sahara Africa and South (east) Asia. These studies employed different indicators in different countries. The type of FS indicator employed in these studies by country is summarised in Fig. 6 (reported only for those countries where at least two indicators were used). The HFSSM indicator was used 7 times in the USA (the highest at country level), which is expected as the HFSSM is used for monitoring household-level food security in the USA. The HDDS was used four times in Kenya whereas the calorie adequacy indicator and HDDS were used three-times each in Ethiopia and Bangladesh.

**Fig. 7:**
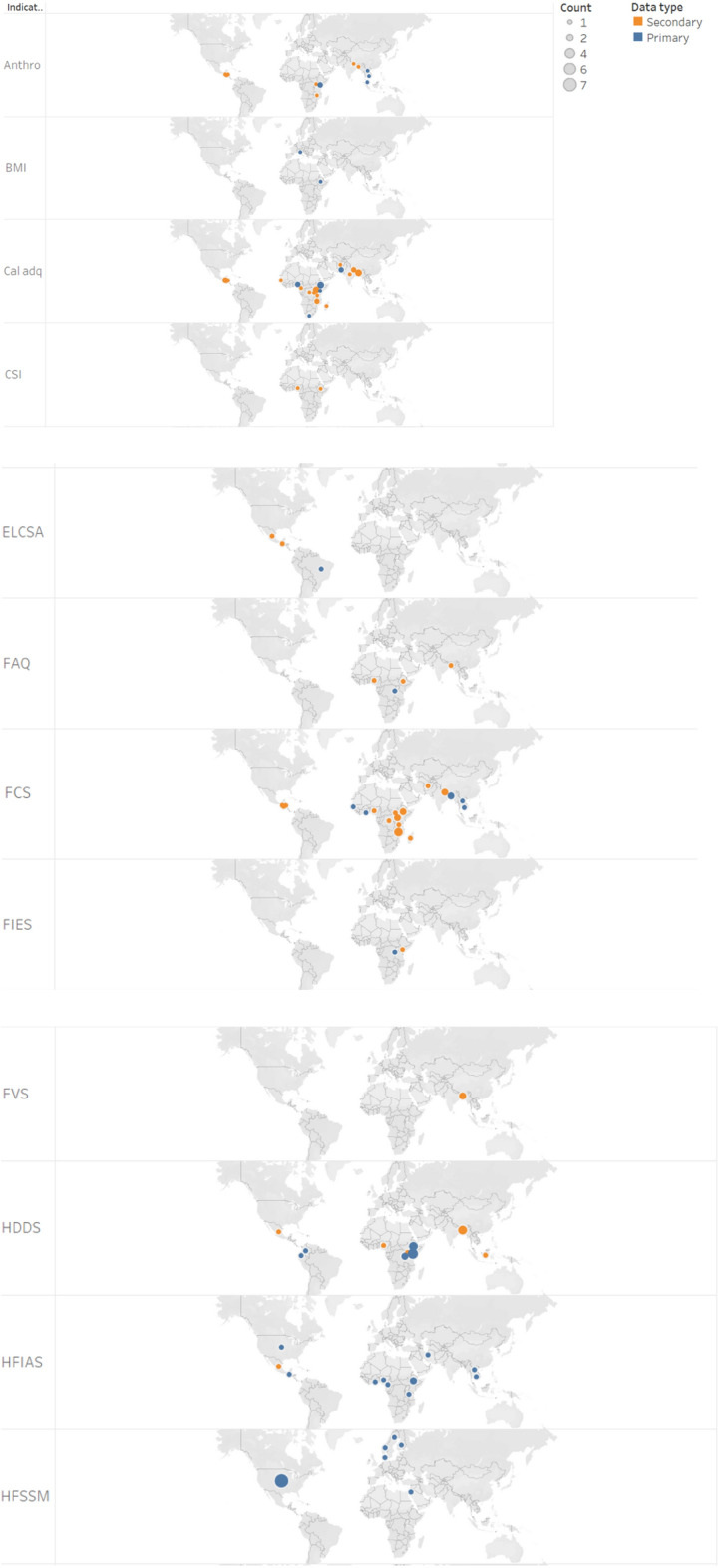

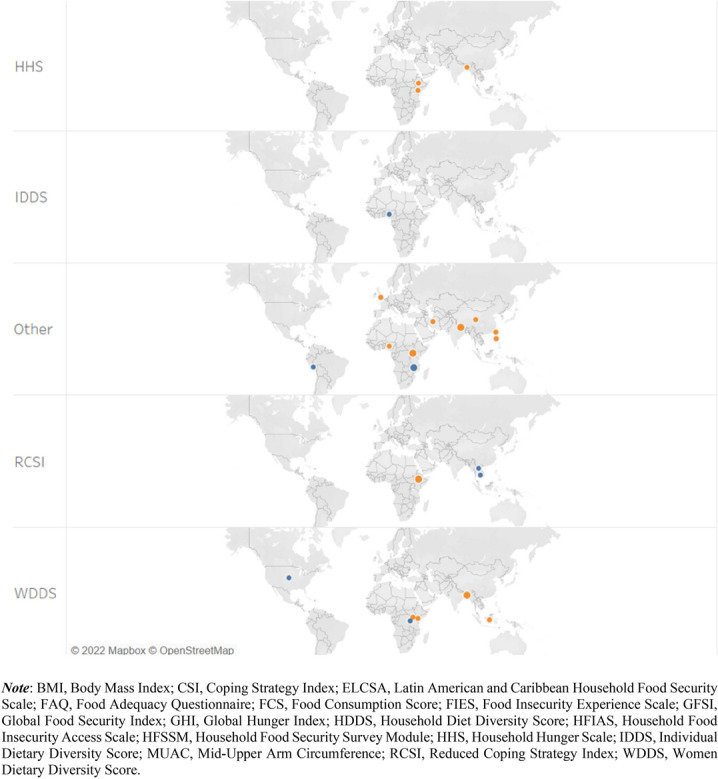
Summary of indicators used by country and data source [Note: Multiple indicators could be used per study, and a study may cover multiple countries]

**Fig. 8:**
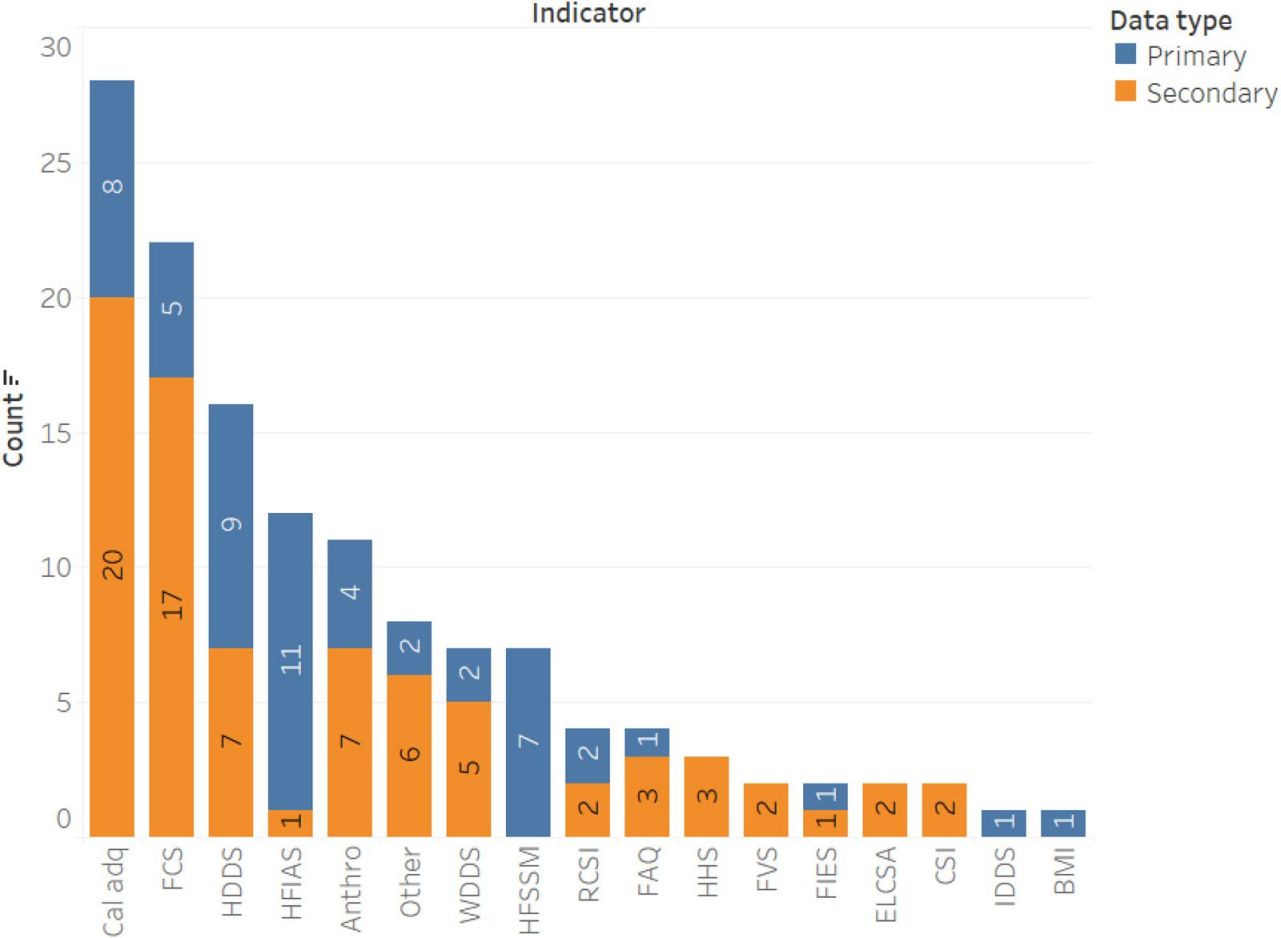
Summary of indicators used by data source [Note: Multiple indicators could be used per study]

About 42% of the 78 studies employed primary data. The majority of these 33 studies applied experience-based indicators: HFIAS (9 articles), HFSSM (6 articles), and other experience-based indicators (4 articles). Dietary diversity-based indicators (12 articles) and calorie adequacy indicator (8 articles) were also applied frequently by studies that employed primary data (Fig. 7). The distributions of the 33 studies that employed primary data by region is as follow: Africa (15 articles), Asia (7 articles), Central and South America (4 articles), Europe (2 articles) and North America (5 articles). The USA and Ethiopia are the countries with the highest number of studies by country (5 and 4 studies, respectively) (Fig. 7). The majority of the studies that applied calorie adequacy indicator and FCS have employed secondary data whereas most of the studies that applied experience-based indicators have employed primary data (Fig. 8). This may imply the fact that collecting data for experience-based indicators is convenient compared to the other type indicators such as the dietary-based ones.

### Quantitative characterization of food security dimensions and components

An ideal food security indicator should capture all the four food security dimensions (availability, access, utilization and stability) and components (quantity, quality, safety and preference). Because ‘measuring food security explicitly’ was one of our inclusion criteria for selecting articles (Fig. 1), and as the most commonly used food security indicators in the literature are measures of food access (Tables 1, 2, 3, 4), all the 78 articles measured the food access dimension. However, the utilisation (13%) and stability (18%) dimensions of food security were seldomly captured. For measuring food utilisation, six of the ten articles applied anthropometry measures [64, 68, 79, 93–96]. Izraelov and Silber [7] applied the Global Food Security Index (GFSI), which allows measuring food utilisation as a construct using 11 indicators. Slimane et al. [56] derived an indicator of food utilisation from ‘*access to improved water sources* and *access to improved sanitation facilities*’, which are two of the ten indicators of the food utilisation dimension in FAO’s Suite of Food Security Index (Table 2; [29]. In the literature, the stability dimension has commonly been captured by using (i) composite indices [7, 12], (ii) the concepts of vulnerability [35, 36, 61, 69, 86] and resilience [74, 88, 90], (iii) econometric approaches [76, 88, 96] (iv) dynamic farm household optimisation model [97], and (v) measuring food security over time/seasons [76, 83].

Almost all the studies analysed the quantity and quality components of food security, whereas the food safety and preference/cultural acceptability components were rarely captured during food security measurements. Although these components are critical in achieving food security according to the 1996 World Food Summit definition of food security, only 2 and 18 studies (out of the 78 articles) captured the food safety and preference components, respectively. Most of the studies (11 articles) that captured the preference component applied the HFIAS indicator, as the second question of the HFIAS 9-items questionnaire addresses the preference food security component. On the other hand, Izraelov and Silber [7] using the GFSI and Ambikapathi et al. [98] using an experience-based food security indicator captured the food safety component.

Only 3 of the 78 publications employed a comprehensive food security measurement, where they measured food security by explicitly considering all the four food security dimensions [7, 12, 96]. Caccavale and Giuffrida [12] and Izraelov and Silber [7] used composite food security indices to capture the four food security dimensions, while Upton et al. [96] applied a moment-based panel data econometric approach to the concept of development resilience in food security measurement. Caccavale and Giuffrida [12] developed the Proteus Composite Index (PCI) for measuring food security at national level. PCI can be used to monitor the food security progresses of countries by comparing within (over time) and between countries. It addresses the shortcomings of other composite indicators in terms of weighting, normalisation, and sensitivity. The PCI is constructed from 21 indicators: availability (2 indicators), access (7 indicators), utilisation (2 indicators), and stability (10 indicators) (Table 5). Eleven of these indicators were adopted from FAO’s Suite of food security Index [30].

**Table 5:** Indicators per food security dimension used in Proteus Composite Index [12]

Dimension	Indicators	Part of suite of food security index [30]
Availability	Average dietary energy supply adequacy (%) (3 year average)	Yes
	Average protein supply (g/capita/day) (3 year average)	Yes
Access	Paved roads over total roads (%)	Yes
	Road density (per 100 square km of land area)	Yes
	Food inflation (headline inflation when not available) (annual %)	No
	GDP, PPP (constant 2011 international $), per capita	Yes
	Remittance inflow, PPP (constant 2011 international $), per capita	No
	Prevalence of undernourishment, share of population	Yes
Utilization	People using at least basic sanitation services (% of population)	Yes
	People using at least basic drinking water services (% of population)	Yes
Stability	Stock-to-use ratio of cereals	No
	Current account balance (current US$), share of GDP	No
	Real effective exchange rate	No
	Value of food imports over total merchandise exports	Yes
	Political stability and absence of violence/terrorism (index)	Yes
	Deaths (two-sided, one-sided, non-state violence), share of total population	No
	People of concern originated (IDPs and refugees), share of total population	No
	People of concern hosted (IDPs and refugees), share of total population	No
	People affected by disasters (10-year weighted), share of total population	No
	Damage from disasters in US$ (10-year weighted), share of GDP	No

Izraelov and Silber [7] is the only study (out of the 78 publications) that applied the GFSI for measuring food security at national level. Like FAO’s Suite of Food Security Index, the GFSI is a composite food security indicator that measures all the four dimensions of food security. Because the GFSI primarily assesses and monitors food security at a national level (i.e. ranking of countries based on the GFSI score), Izraelov and Silber [7] investigated the sensitiveness of the rankings of countries to the list of indicators used for the different dimensions and to the set of weights elicited from the panel of experts of the Economic Intelligence Unit by employing PCA and/or data envelopment analysis (DEA) methods. The authors concluded that the rankings based on the GFSI are robust in relation to both the expert weights used and the choice of indicators. The Economist Intelligence Unit (EIU) (2021) produces the GFSI index each year by using 69 indicators covering the four dimensions of food security: availability, affordability (accessibility), quality and safety (utilization), and natural resources and resilience (stability).

Upton et al.’s [96] defined four axioms that an ideal food security measure must reflect. Relying on the 1996 World Food Summit food security definition [4], they defined the following four axioms:

- Scale axiom: it addresses both individuals and households at different scale of aggregation (e.g. regions) reflecting ‘all people’;
- Time axiom: reflecting ‘at all times’, it captures the food stability dimension to account for both predictable and unpredictable variability of food security over time;
- Access axiom: derived from ‘physical, social and economic access’, it captures the food access (and implicitly the availability) dimensions; and
- Outcomes axiom: reflecting on “an active and healthy life”, it reflects the food utilization dimension, which captures the dietary, nutrition, and/or health outcomes.

Upton et al. [96] did note that no food security measure at the time satisfied all these four axioms in the literature. In response, they employed a stochastic dynamic measure of well-being based on the concept of development resilience [99]. Barrett and Constas [99] defined development resilience as ‘the capacity over time of a person/household... to avoid poverty in the face of various stressors and in the wake of myriad shocks. If and only if that capacity is and remains high over time, then the unit is resilient’ (p. 14). [100, 101] demonstrated the econometric implementation of the stochastic dynamic measure of well-being at multiple scales using household or individual survey data. They showed how a measure of household or individual well-being and resilience can be estimated, and aggregated at regional or national level using a system of conditional moment functions. By adopting the [100, 101] moments-based (dynamic) panel data econometric approach, Upton et al. [96] used the resilience concept in food security measurement to reflect the above four axioms as follows:

- The scale axiom is satisfied by estimating food security at the individual or household level, and then by aggregating it into higher-level groups (e.g. regions).
- The time/stability axiom is captured by using [100, 101] dynamic approach.
- The access axiom is considered by conditioning the moments of the food security distribution regarding economic, physical, and social factors that influence food access.
- The outcome (utilisation) axiom is considered by using nutritional status indicators as dependent variables in the econometric model. Upton et al. [96] used HDDS and child MUAC as outcome indicators.

### Recent developments in food security measurement

The concepts of vulnerability and resilience have only recently been introduced in food security measurement and analysis. Rather than directly measuring food security or food insecurity, researchers have been seeking to measure vulnerability to food insecurity and food security resilience, and their respective determinants/drivers. Out of the 78 publications, 5 and 4 articles respectively employed the concepts of vulnerability [35, 36, 61, 69, 86] and resilience [74, 88, 90, 96] in their food security measurement and analysis.

Ibok et al. [36] developed the Vulnerability to Food Insecurity Index (VFII) for measuring the vulnerability of households to food insecurity, and validated it by comparing the estimates of vulnerability to food insecurity with the traditional food insecurity measures (calorie adequacy, CSI, FCS). The VFII is a composite index constructed from three dimensions (Table 6): exposure (probability of covariate shock occurring), sensitivity (previous/accumulative experience of food insecurity), and adaptive capacity (how households respond, exploit opportunities, resist or recover from food insecurity shocks, which is the coping ability of households). A set of indicators are used for each of the three dimensions (Table 6). By defining thresholds, Ibok et al. [36] assigned households into one of the three categories: highly vulnerable, mildly vulnerable, and not vulnerable to food insecurity. The results showed that VFII has a weak positive correlation with FCS and per capita calorie adequacy, whereas it has a negative correlation with CSI. Some of the households with poor calorie per capita consumption were classified as not vulnerable to food insecurity, whereas some households with acceptable calorie per capita consumption were identified as highly vulnerable to food insecurity. The authors concluded that a household’s vulnerability to food insecurity can be better measured using CSI than using FCS and per capita calorie adequacy (using the VFII as a benchmark).

**Table 6:** Indicators used for constructing the vulnerability to food insecurity index [36]

Dimension	Indicators	Description of variables
Exposure	Health shock	Illness of income earning member
	Unemployment shock	Job loss
	Civil conflict shock	Theft of crops, cash, livestock or other Kidnapping/Hijacking/robbery/assault
	Agro-climatic shock	Poor rain that caused harvest failure Flooding that caused harvest failure
	Food price shock	Increase in price of major food items consumed
Sensitivity	Malnutrition	Length/height-for-age (stunting)
	Child mortality	Total number of children dead in each household
	Hunger	Number of days’ households gone without eating any food
Adaptive capacity	Wealth Index	Household assets used to assess information Mobility assets used in households Livelihood assets own by households Housing structure characteristics
	Access to infrastructure	Household distance to nearest major road (km) Household distance to nearest market (km) Time taken to walk from home to water source (minutes)
	Livelihood activities	Income from savings, rental of properties and other sources Estimated revenue from non-farm enterprises Total yield of crops harvested (kg)
	Household literacy	Years of schooling for household heads

[86] analysed the effects of households’ vulnerability to different climatic hazards on their food access by employing a generalised linear regression model. They used FCS as a measure of household food access, concluding that households that are vulnerable to flood were found to be more likely to be food insecure (i.e. to have a low FCS) than less vulnerable households.

Vaitla et al. [88] and Upton et al. [96] employed dynamic panel data modelling to measure the food security resilience of households. They analysed the determinants of food security status at a point in time, and its food security resilience by using different food security indicators. They defined resilience as ‘the probability that a household is truly above a chosen food security cut-off, given its underlying assets, demographic characteristics, and past food security status’. Similar to Upton et al. [96], they used the moments (mean and variance) of the food security score over time to estimate resilience as the probability of attaining a given level of food security. Vaitla et al. [88] used FCS and RCSI as a dependent variable in their dynamic panel data model. They concluded that the determinants of a household’s food security status and food security resilience are different. They also showed that the drivers of food security resilience vary across the two food security measures used as dependent variables.

Lascano Galarza [90] investigated the effects of food assistance on a household’s food security status at a point in time, and its food security resilience, by applying FAO’s Resilience Index Measurement and Analysis II framework. The author used FCS and food expenditure as measures of food security when evaluating the effects of the food assistance program and the household’s resilience on food security status. Factor analysis and multiple indicators multiple causes models were used to construct the resilience score and to analyse its effect on food security. The resilience score was derived from four indicators: assets, access to basic services, social safety nets, and adaptive capacity. The author ultimately found a significant positive association of food assistance programmes with a household’s food security status and food security resilience.

Smith and Frankenberger [74] analysed the effects of resilience capacity in reducing the effect of shocks on household food security using HHS and FAQ (number of months of inadequate household food access) as measures of food security. The results of their fixed effect panel data model showed that resilience capacity enhancing attributes such as household assets, human capital, social capital, information access, women empowerment, diversity of livelihood, safety nets, and market access reduce the negative effect of flooding on household food security.

## Discussion

### Which food security indicator is the best?

**Fig. 9:**
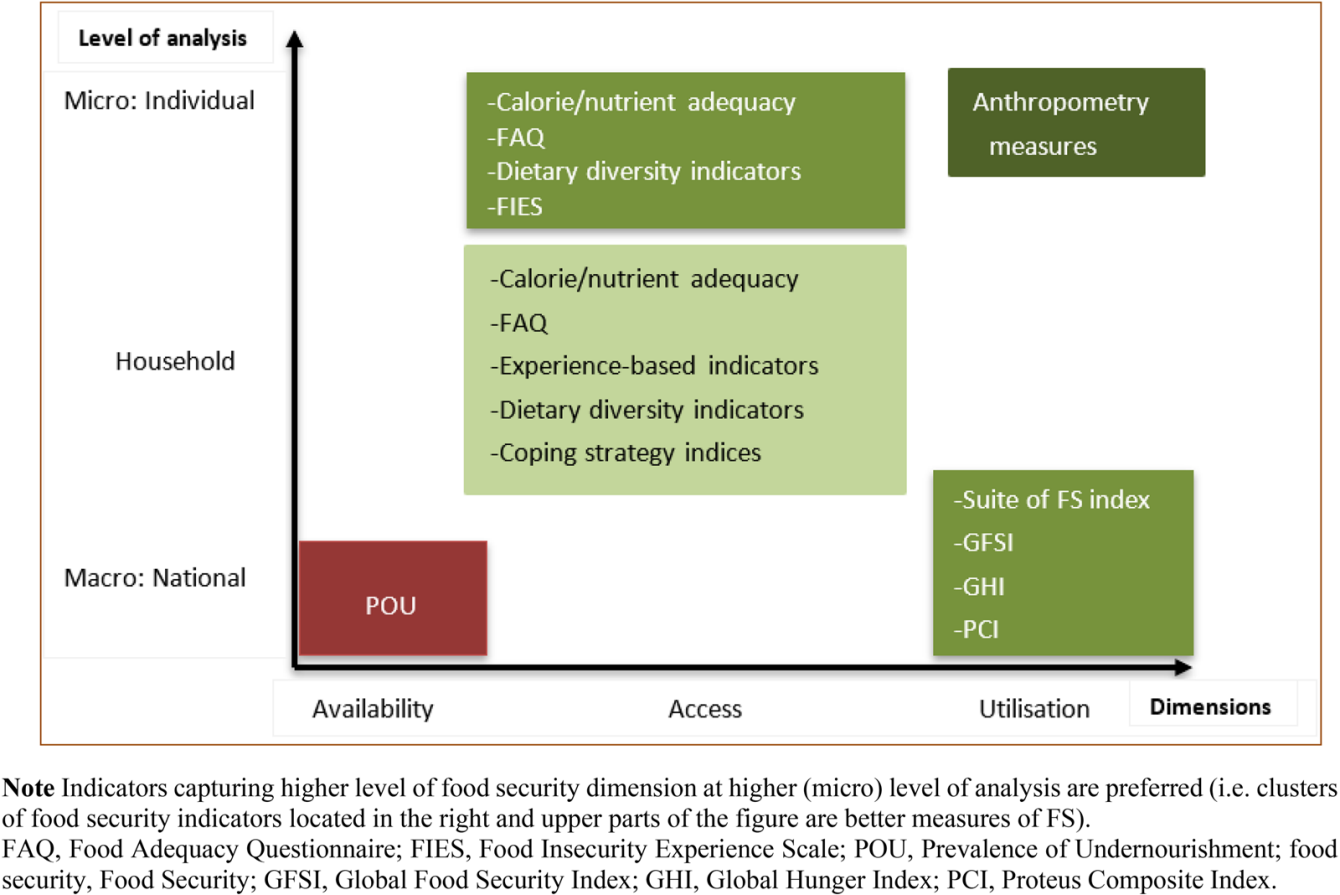
Summary of the retrieved indicators according to the level of analysis and food security dimensions

Although numerous food security indicators have been developed for use in research, there is no agreement on the single ‘best’ food security indicator among scientists or practitioners for measuring, analysing, and monitoring food security [9, 12]. The different international agencies also use their own sets of food security indicators (e.g. World Food Programme: FCS, USAID: HFIAS; FAO: POU and FIES; and EIU: GFSI). Figure 9 summarises the most applied food security indicators according to the level of analysis and the food security dimensions that they intend to reflect. The level of analysis ranges from macro (e.g. national) to micro (e.g. individual) levels, and the measured food security dimension from availability to utilisation. An ideal food security indicator should capture all the four food security dimensions at individual level to reflect the 1996 World Food Summit definition of food security. However, most of the available indicators are measures of food access at the household level (Fig. 9). Only a few composite and anthropometry indicators can measure food utilisation (besides availability and access) at national and individual levels, respectively. On the other hand, the stability dimension can be captured by estimating food security indicators over time or as described above in ‘‘Quantitative characterization of food security dimensions and components’’ Sect. The three composite indicators GFSI [26], Suite of Food Security Index [29], and PCI [12] can allow to directly measure the stability dimension of food security while also capturing the other three food security dimensions at national level.

In general, there exist an inherent trade-off when choosing one indicator over another type of indicator because the various classes of food security indicators reflect different aspects of food security [96] such as dimensions, components, levels of analysis (e.g. national vs individual), and data requirement (subjective vs objective; recall period of 1 year vs 24 h). Therefore, most of the commonly used indicators can be considered as mutually complementary than substitutes for one another. The subjective experience-based indicators, for example, measure a household’s experience of anxiety/worry/hunger arising from lack of food access, whereas the objective dietary diversity-based indicators measure a household’s access to diverse food, reflecting a household’s caloric intake and diet quality. Household dietary diversity-based and caloric adequacy indicators also complement each other because sufficient calorie might be achieved with low food quality (without diversified diet), whereas a diverse diet might not be enough to meet a household’s caloric requirement. Noting this complementarity, Bolarinwa et al. [76] classified households into three categories of food insecurity (food secure, partially food insecure, and completely food insecure) by integrating two indicators: HDDS and per capita food expenditure (where the food expenditure reflects caloric adequacy).

### Data requirements of food security measurement

**Fig. 10:**
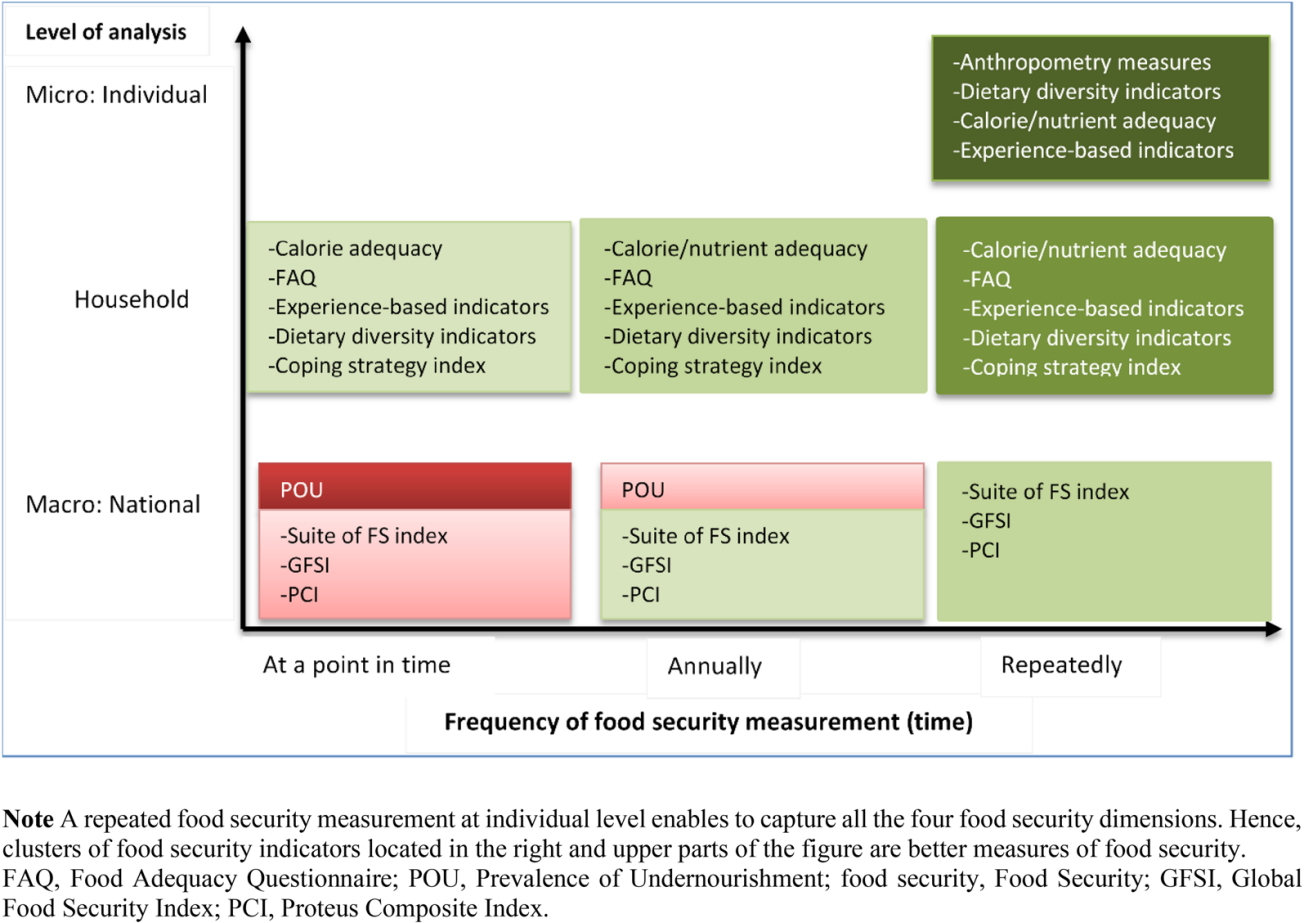
High frequency food security measurement for better food security analysis.

The most critical challenge of a comprehensive food security measurement and analysis is generating reliable data consistently for estimating complementary food security indicators (at the individual level) [13]. Measuring food security with a high frequency consistently over time (e.g. quarterly instead of annually) at the individual level by applying a set of complementary indicators (e.g. calorie/nutrient adequacy and anthropometry measures) can help us better analyse and monitor food security (Fig. 10). A national level food security measurement at a point in time (e.g. using POU) is less informative for decision-making compared with measuring food security every year (or ideally in real-time) at the household level (e.g. using calorie adequacy). Integrating food consumption and anthropometry information in regular national household living standard surveys can also be crucial to eliminating the limitations of current measurement approaches, especially because nutrition, food consumption, health, and income are interrelated [13].

De Haen et al. [13] rightly remind us that to improve the reliability and accuracy of a nation’s food security measurement and analysis, ‘the focus should be on generating more timely, comprehensive, and consistent household surveys that cover food consumption and anthropometry, [which] allow much better assessment of the prevalence of food insecurity and undernutrition, as well as of trends and driving forces.’ That is, first, generating data from a nationally representative sample through comprehensive household surveys allows us to estimate a set of complementary indicators reflecting the different aspects of food security measurement (dimensions, components, outcomes, behavioural responses, coping mechanisms) (Fig. 10). Second, comprehensive surveys help measure both the prevalence of food insecurity and its drivers/determinants. Third, it is critical to generate these data consistently over time so that the progress towards food security can be monitored, drivers can be analysed over time, and food insecurity can be detected well in advance. This approach could address the UN Scientific Group’s criticism [11] that ‘existing early warning systems lack indicators to adequately monitor degradation of food systems.’ Fourth, the data allow us to analyse and evaluate the effects of programmes and interventions (over time) at different levels (individual, household, and national). It also opens opportunities to conduct development research in food, nutrition, health, and poverty [13].

In summary, we suggest the following points in the light of the above discussions for a comprehensive food security measurement:

- Food security should be measured at the individual (or at least at household) level by applying a set of complementary food security indicators to capture the availability, access, and utilisation dimensions of food security. Combining anthropometry measures with other objective food security indicators (e.g. calorie adequacy or dietary diversity indicators) will further allow us to capture these three dimensions.
- The fourth dimension of food security, i.e. the stability dimension, can be captured by producing the estimates of the complementary food security indicators over time or in real time. A repeated high frequency food security measurement (if possible by using near real-time data) is thus preferable, as it can also help to identify the onset of food insecurity in time, to evaluate interventions/programs, and to monitor food security progresses.
- The behavioural aspects of food insecurity and the cultural acceptability of food can be measured by using one of the experience-based measures. For example, FAO’s FIES can be applied to estimate the prevalence and severity of food insecurity at individual level. Because the FIES has been applied in more than 100 countries, countries can compare their respective food security states with each other.
- The use of experience-based indicators (e.g. FIES) allows conducting rapid food security assessments as the data collection is easier compared to the objective food security indicators (e.g. calorie adequacy).
- Integrating food consumption (intake, expenditure, and diet diversity) and anthropometry information in regular national household living standard surveys enables us to collect complete and consistent data for estimating complementary food security indicators in food security analyses.

### Study limitations and future research

In this study, we identified and characterized the most commonly applied food security indicators in the literature with respect to the dimensions and components covered, methods and models of measurement, level of analysis, data requirements and sources, intended purpose of application, and strengths and weaknesses. Subsequently, we analysed data on food security measurement from 78 peer-reviewed articles, and suggested the estimation of complementary food security indicators consistently over time for conducting a comprehensive analysis by taking all the four food security dimensions and components into account. In order to select the set of these complementary food security indicators that would be applicable to a specific context (e.g. country or region), we recommend to conduct a Delphi study by involving food security experts, policy-makers and other relevant stakeholders. In addition, we limited the literature search to two databases (Scopus and WoS) and included only peer-reviewed articles in this study. Therefore, we suggest to extend this study by broadening the literature type by including the grey literature (e.g. reports, book chapters and conference proceedings) and by searching from other databases, which reduce the publication bias. Moreover, we followed the 1996 World Food Summit definition of food security [5], which provided the foundation for the four food security dimensions (*availability*, *access*, *utilisation*, and *stability*). Accordingly, in this study, we organised the literature review on food security measurement over these four dimensions. However, food system researchers have recently noted the need to update the definition of food security in reference to sustainable food systems, for example, by including new food security dimensions [102–104]. Clapp et al. [103], for example, proposed the inclusion of two extra dimensions (*sustainability* and *agency*) to improve the framework of food security analyses. The inclusion of these two extra dimensions guarantees that every human being has access to healthy and nutritious food, not only now but also in the future. In this regard, sustainability can be considered as a pre-requisite for long-term food security [103, 104]. Therefore, we recommend future research to operationalize literature reviews according to the six food security dimensions (i.e. *availability*, *access*, *utilisation*, *stability*, *sustainability* and *agency*). Furthermore, most existing studies about food security measurement in the literature are based on the 1996 World Food Summit definition of food security [5]. Food security analyses based on this definition narrows the scope of the food security concept, and do not support system level analysis by considering other components of the food system. For example, food security is a subset (component) of the Food Systems Approach, which takes food environments, food supply chains, individual factors, external food system drivers, consumer behaviour, and food system outcomes (e.g. food security and health outcomes) into account [105–108]. Therefore, given the increasing attention to the Food Systems Approach and system level analyses in the literature, the Food Systems Approach can be used as a framework for operationalising future literature reviews on food security.

## Conclusion

We critically reviewed numerous food security indicators and methodologies published in scientific articles since the last decade using the SLR methodology. We reviewed, analysed, and summarised the results of 78 articles on food security measurement. We found that the household-level calorie adequacy measure was the most frequently used indicator in the literature as a sole measure of food security. Dietary diversity indicators (HDDS, WDDS, IDDS, and FCS) and experience-based indicators (HFSSM, FIES, HFIAS, HHS, ELCSA) were almost equally in use and popular. In terms of the food security dimensions, food utilisation (13%) and stability (18%) were seldom captured. Caccavale and Giuffrida [12], Izraelov and Silber [7], and Upton et al. [96] are the only studies that measured food security by considering all four dimensions. We also found that the majority of the studies that applied calorie adequacy and dietary diversity-based indicators employed secondary data whereas most of the studies that applied experience-based indicators employed primary data, suggesting the convenience/simplicity of collecting data for experience-based indicators than dietary-based indicators. The use of experience-based indicators allows conducting rapid food security assessments whereas the use of complementary indicators is required for food security monitoring over time. We conclude that the use of complementary food security indicators, instead a single indicator, better capture the different food security dimensions and components,this approach is also beneficial for analyses at different levels. The results of this study, specifically the analysis on data requirements for food security measurement, can be used by food security stakeholders such as governments, practitioners and academics for briefs, teaching, as well as policy-related interventions and evaluations.

## Supplementary Information

**Additional file 1:**  Data and list of articles used in the systematic literature review on food security measurement (N = 78).

**Additional file 2: Table S1.** Summary of the publications that applied dietary diversity score indicators. **Table S2.** Summary of the publications that used Food Consumption Score (FCS). **Table S3.** Summary of the publications that used HFIAS and HHS. **Table S4.** Summary of the publications that used HFSSM and ELCSA. **Table S5.** Summary of the publications that used FIES.

## Data Availability

All data are available within the paper.

## Footnotes

1 Detailed discussion on this issue can be found in''Which food security indicator is the best?'' Sect.

2 In Scopus, since the research field ‘Agricultural and Biological Sciences’ domain is very broad, we excluded studies in the areas of biology, chemistry, ecology, environment, forestry, aquaculture, and plant/crop sciences during the literature search (via “AND NOT”).

3 In line with this, our final food security measurement dataset does not contain articles from 2010 Additional file 1.

4 The call to the special issue can be retrieved from the journal’s website: https://www.sciencedirect.com/journal/global-food-security/special-issue/10F642R6J6K.

5 This confirms the lack of due attention given to the standardization and harmonisation of food security measurement prior to 2010, and the lack of consensus among researchers, practitioners, or governments on the indicators and methodologies that should be applied for measuring and monitoring food security.
