# Supplementary material for: A systematic literature review of indicators measuring food security
Source: Agric Food Secur. 2023 May 5;12(1):10. doi: 10.1186/s40066-023-00415-7 (PMC10161169; doi:10.1186/s40066-023-00415-7)
Supplement: Supplementary file 2 — Additional file 2: Table S1. Summary of the publications that applied dietary diversity score indicators. Table S2. Summary of the publications that used Food Consumption Score (FCS). Table S3. Summary of the publications that used HFIAS and HHS. Table S4. Summary of the publications that used HFSSM and ELCSA. Table S5. Summary of the publications that used FIES. [file 40066_2023_415_MOESM2_ESM.docx]

**A systematic literature review of indicators measuring food security**

**Supplementary Material B: Summary of articles by the type of food security indicator used**

**Table S1** Summary of the publications that applied dietary diversity score indicators

| Article | Objective of the study | Intended use | Recall  period | Other FS indicators used | Data | Method |
| --- | --- | --- | --- | --- | --- | --- |
| Tambo *et al.* (2021) | Assessing the effect of provision of plant health services on food insecurity prevalence among farming households in Rwanda | Program evaluation (HDDS; WDDS) | 24 hours | FIES; FAQ | Survey: 637 households (263 users and 374 non- users) | Endogenous switching regression model; propensity score matching |
| Bakhtsiyarava *et al.* (2021) | Analysing (agricultural and environmental) determinants of household-level food security | Cause–effect analysis | 7 days | FAQ | LSMS (Ethiopia: 2960/2809 in 2014/2016; Nigeria: 3055/2954 in 2013/2016) | Logistic and non-parametric (random forest) regression models |
| Islam *et al.* (2018) | Assessing the impact of farm diversification on household diet diversity | Cause–effect analysis: HDDS/WDDS | 7 days | FVS | Bangladesh Integrated Household Survey | Conditional fixed effect; Poisson model |
| Bolarinwa *et al.* (2020) | Analysing the dynamics of household food security and its determinants across agricultural seasons | Cause–effect analysis | 1 month | Per capita food expenditure | National Institutes of Statistics of Rwanda (14,419 households) | Correlated random effects multinomial logit model |
| Sibhatu & Qaim (2018) | Analysing the relationship between production diversity and dietary quality in farm households and the mechanisms | Cause–effect analysis | 7 days | WDDS, calorie and micronutrient adequacy | Farm household survey in Indonesia (672), Kenya (393), and Uganda (417) | OLS with robust standard errors |
| Maxwell *et al.* (2014) | Comparing food insecurity prevalence estimates of the commonly used food security indicators over time | Prevalence estimation | 24 hours | CSI; RCSI; HFIAS; HHS; FCS | Survey: panel data from 300 households (two years’ semi-annual data) in Ethiopia | (Dynamic) correlation analysis |
| Upton *et al.* (2016) | Analysing the prevalence and determinants of food insecurity over time | Prevalence and determinants of FI | 7 days | Child MUAC | ILRI (from 924 households over 2009–2013 from Kenya) | Moments-based panel data econometric approach |
| Islam *et al.* (2016) | Analysing the effect of microcredit programme participation on household food security | Program evaluation | 7 days | Calorie adequacy; FVS; child/women arthrometry | Survey (four rounds): 3,000 households | Propensity score matching |
| Chege *et al.* (2016) | Assessing the food security and nutritional status of children under five years in households affected by HIV | FI prevalence estimation | 7 days | Child arthrometry | Survey: 286 households | Descriptive statistics (correlation analysis) |
| Hussein *et al.* (2018) | Evaluating the reliability of household food security indicators (HFIAS and HDDS) for assessing nutritional status of individuals (BMI) in clinical context | Prevalence estimation | 24 hours | HFIAS, BMI | Survey: 423 individuals in clinical situation in Ethiopia | Cronbach’s alpha; receiver operating characteristic curves |
| Vellema *et al.* (2016) | Verifying the construct validity of HDDS | Prevalence estimation | 24 hours | - | Survey: 1,015 households in Colombia and Ecuador | Rasch model |
| Fawole *et al.* (2016) | Assessing the food security status of households in Nigeria | Prevalence estimation | 24 hours | Calorie adequacy | Survey: 150 households | Descriptive analysis |
| Hossain *et al.* (2019) | Evaluating the performance of household characteristics and dietary diversity indicators in predicting calorie intake | Prevalence estimation | 7 days | WDDS; FCS | 6,427 households [Bangladesh Integrated Household Survey] | Machine learning and  econometric methods |
| Nicholson *et al.* (2021) | Analysing food security while considering all the four dimensions using agricultural systems models | Prevalence estimation; cause–effect analysis | 24 hours | FIES/HFIAS and food consumption expenditures | Data on farm inputs, outputs, and household food access indicators and determinants | Dynamic farm household optimisation model |
| Abu & Oldewage-Theron (2019) | Assessing food insecurity among college students | Prevalence estimation (WDDS) | 24 hours | HFIAS | Survey: 173 college students in the US | ANOVA; odds ratio |
| Sholeye *et al.* (2019) | Assessing food security and dietary diversity among individuals | Prevalence estimation | 24 hours | HFIAS | Survey: 150 individuals in Nigeria | Descriptive analysis |

***Note***: FS, Food Security; FI, Food Insecurity; BMI, Body Mass Index; CSI, Coping Strategy Index; ELCSA, Latin American and Caribbean Household Food Security Scale; FAQ, Food Adequacy Questionnaire; FCS, Food Consumption Score; FIES, Food Insecurity Experience Scale; GFSI, Global Food Security Index; GHI, Global Hunger Index; HDDS, Household Diet Diversity Score; HFIAS, Household Food Insecurity Access Scale; HFSSM, Household Food Security Survey Module; HHS, Household Hunger Scale; IDDS, Individual Dietary Diversity Score; MUAC, Mid-Upper Arm Circumference; RCSI, Reduced Coping Strategy Index; WDDS, Women Dietary Diversity Score.

**Table S2** Summary of the publications that used Food Consumption Score (FCS)

| Article | Objective of the study | Purpose of FCS use | Other food security indicators used | Data | Method |
| --- | --- | --- | --- | --- | --- |
| D'Souza and Jolliffe (2014) | Analysing the effect of staple food price shocks on the food security status of vulnerable households. | Cause–effect analysis: FCS, as a measure of dietary diversity, was used to capture ‘food utilisation’ | Per capita daily caloric intake | Survey data from 20,483 households in Afghanistan | Unconditional quantile regression |
| Maxwell *et al.* (2014) | Comparing food insecurity prevalence estimates of the commonly used food security indicators over time | Food insecurity prevalence estimation: FCS is assumed to have a positive correlation with calorie intake | CSI, RCSI, HDDS, HFIAS, HHS | Household survey (300) panel data (two years’ semi-annual data) in Ethiopia | (Dynamic) correlation analysis |
| Bühler *et al.* (2018) | Understanding the association between household’s food security status and individual’s nutritional outcomes | Food insecurity prevalence estimation: FCS was used to capture food access and to some extent food utilisation as it relates with food quantity and quality | RCSI, HFIAS, child stunning, wasting, and underweight | Survey data from 1,200 rural households in Cambodia and Lao PDR | Regression analysis (OLS and probit models) |
| Hossain *et al.* (2019) | Evaluating the performance of household characteristics and dietary diversity indicators in predicting calorie intake | Food insecurity prevalence estimation: proxy for household calorie intake | HDDS, WDDS | 6,427 households [Bangladesh Integrated Household Survey] | Machine learning and  econometric methods (e.g. random forest) |
| Lokosang *et al.* (2011) | Exploring the application of various ordinal logistic regression methods in predicting food security | Determinants of food security for monitoring and early warning: FCS is used as a food security indicator in analysing the determinants of food security | - | 9,220 households in South Sudan [Sudan Household Health Survey] | Ordinal logistic regression |
| Hjelm *et al.* (2016) | Examining the association between wealth indices and food security indicators | Food insecurity prevalence estimation: measuring household diet quality | Calorie adequacy, share of expenditure on food, share of calories from staples | National household surveys from five countries in Africa and Asia | Descriptive analysis; correlation analysis |
| Ibok *et al.* (2019) | Developing an index for measuring the vulnerability of households to food insecurity, and comparing it with traditional food security measures | Food insecurity prevalence estimation: to compare the prevalence estimates from FCS and the new index | CSI, calorie adequacy, Vulnerability to Food Insecurity Index | Living Standard Measurement Study’s panel data from 5,000 households in Nigeria | Descriptive analysis; correlation analysis |
| Vaitla *et al.* (2020) | Analysing the determinants of food security resilience by using different food security indicators | Cause–effect analysis and monitoring: FCS measures household diet diversity and RCSI the behavioural response | RCSI | Household survey (300) panel data (four surveys over 18 months) | OLS and generalized linear models |
| Lovon and Mathiassen (2014) | Evaluating the relationship between food insecurity estimates of FCS and household food insecurity estimates based on calorie adequacy | Food insecurity prevalence estimation: FCS is used as a proxy for household diet quantity and quality | Calorie adequacy (three classes), Child stunning | Household survey data from six countries | Descriptive analysis; correlation analysis |
| Marivoet *et al.* (2019) ^†^ | Assessing the validity of FCS in capturing diet quantity, quality, and adequacy across regions, and evaluating the standard FCS thresholds | Food insecurity prevalence estimation: FCS is used as a measure of household food access | Calorie and micronutrient adequacy | HCES data from 12,097 households in the Democratic Republic of the Congo (DRC) | Descriptive, regression, and correlation analysis |
| Tuholske *et al.* (2020) | Comparing household food insecurity prevalence estimates of FCS and HFIAS, and their determinants | Food insecurity prevalence estimation and cause–effect analysis: FCS is used as a measure of household food access | HFIAS | Urban household survey (668) in Accra, Ghana | Descriptive, correlation, and regression analyses |
| Dibba *et al.* (2017) | Analysing the effect of adoption of a new rice variety on household FS | Impact evaluation: FCS as a measure of food availability, access, and utilisation | Number of household sick days per capita | Survey data from 502 rice farming households  in The Gambia | Instrumental variable estimation |
| Islam and Mamun (2020) | Understanding the effects of vulnerability to climatic hazards on household food access | Cause–effect analysis: FCS as a measure of household food access | - | Survey data from 421 households in Bangladesh | Generalised linear regression model |
| Lascano Galarza (2020) | Investigating the effects of food assistance on a household’s resilience to food insecurity and food security status | Impact evaluation: FCS as measure of household wellbeing | Resilience Capacity Index, food consumption expenditure | Survey data from 1,696 households in Malawi | Resilience Index Measurement and Analysis; propensity score matching |

^†^ The FCS was derived using data from a 24-hour recall period unlike the other studies that used 7-days recall period.

***Note***: Refer to the footnotes of Table S2 for the abbreviations.

**Table S3** Summary of the publications that used HFIAS and HHS

| Indicator | Article | Objective of the study | Purpose of HFIAS/HHS use | Other FS indicators used | Data | Method |
| --- | --- | --- | --- | --- | --- | --- |
| HFIAS | Tuholske *et al.* (2020) | Comparing household food insecurity prevalence estimates of FCS and HFIAS, and their determinants | Prevalence estimation; cause–effect analysis | FCS | Survey: 668 urban households in Accra, Ghana | Descriptive, correlation, and regression analyses |
| HFIAS | Chegere *et al.* (2020) | Analysing the effects of hermetic storage bag supply and training on food security by comparing treatment and control groups | Impact assessment (program evaluation) | - | Survey: 390 small-scale maize farmers in Tanzania | OLS estimation with bootstrapped standard errors |
| HFIAS | Ngome *et al.* (2019) | Assessing the relationship between food insecurity prevalence and deforestation | Prevalence estimation; cause–effect analysis | - | Household survey data in Cameroon | Chi-square; ANOVA |
| HFIAS | Abu & Oldewage-Theron (2019) | Assessing food insecurity among college students | Prevalence estimation | WDDS | Survey: 173 students (Texas, USA) | ANOVA; odds ratio |
| HFIAS | Beveridge *et al.* (2019) | Analysing the drivers of food insecurity in Guatemala’s dry corridor region | Prevalence estimation; cause–effect analysis | - | Survey: 195 households, in-depth interview: 25 households | Logistic multiple regression |
| HFIAS | Sholeye *et al.* (2019) | Assessing food security and dietary diversity among adult patients in Nigeria | Prevalence estimation | IDDS | Survey: 150 clients of primary health-care services | Descriptive analysis |
| HFIAS | Bühler *et al.* (2018) | Understanding associations between household’s food security status and individual’s nutritional outcomes | Prevalence estimation | FCS, RCSI, & Child arthrometry | Survey: 1200 households in Cambodia and Lao PDR | Regression analysis (OLS and probit models) |
| HFIAS | Hussein *et al.* (2018) | Evaluating the reliability of household food security indicators (HFIAS, HDDS) for assessing nutritional status of individuals (BMI) | Prevalence estimation | HDDS, BMI | Survey: 423 individuals in clinical situation in Ethiopia | Cronbach’s alpha; receiver operating characteristic curves |
| HFIAS | Salarkia *et al.* (2014) | Assessing the validity and reliability of HFIAS in measuring household food insecurity in the urban areas in Iran | Prevalence estimation | - | Survey: 400 households in Iran | Descriptive analysis |
| HFIAS | Nicholson *et al.* (2021) | Analysing food security while considering all the four dimensions using agricultural systems models | Prevalence estimation, cause–effect analysis | HDDS, FIES, food consumption expenditure | Data on farm inputs, outputs, and household food access indicators and determinants (over time) | Dynamic farm household optimisation model |
| HFIAS;  HHS | Maxwell *et al.* (2014) | Comparing food insecurity prevalence estimates of the commonly used food security indicators over time (seasons) | Prevalence estimation | CSI, RCSI, FCS, HDDS | Household survey (300) panel data (two years’ semi-annual data) in Ethiopia | (Dynamic) correlation analysis |
| Adapted HHS ^†^ | Mutisya *et al.* (2016) | Analysing the effect of education on household food security in Kenya | Cause–effect analysis | - | Panel data: 23,549 households over 2007–2012 in Kenya | Random effects generalised ordered probit model |
| HHS | Smith and Frankenberger (2018) | Understanding the role of resilience capacity in reducing the effect of shocks on household food security | Cause–effect analysis | FAQ | Two rounds of surveys (2012, 2014): 8,415 households in Bangladesh; panel data were available for the 358 households | Ordinary Least Squares with fixed effects; fixed-effect panel data models |

^†^ Mutisya *et al.* (2016) derived HHS from a four-item, instead of the standard three-item, questionnaire following the Radimer *et al.* (1992) approach of measuring hunger and food security.

***Note***: Refer to the footnotes of Table S2 for the abbreviations.

**Table S4** Summary of the publications that used HFSSM and ELCSA

| Indicator | Article | Objective of the study | Purpose of HFSSM/ ELCSA use | Data | Method |
| --- | --- | --- | --- | --- | --- |
| HFSSM | Ahn and Norwood (2021) | Assessing household food security status during the COVID-19 pandemic | Prevalence estimation | Survey: 1,047 household in the US | Descriptive analysis |
| HFSSM | Ahn *et al.* (2020) | Evaluating if food insecurity rates derived from internet sample surveys could approximate food insecurity rates derived from the nationally representative sample in the US | Prevalence estimation | Household data from three internet surveys | Descriptive analysis; Rasch model |
| HFSSM | Burke *et al.* (2020) ^†^ | Evaluating school-based nutrition assistance programs on child food security (by comparing control and treatment groups) | Program evaluation | RCT: 1,393 treatment households and 1,243 control households | Randomized controlled trial; logistic regressions |
| HFSSM | Wright *et al.* (2020) | Assessing dietary quality and intake of under-consumed nutrients among Food Pantry Clients | Prevalence estimation: cause–effect analysis | Data from 579 food pantry adult clients in the midwestern US | Descriptive analysis |
| HFSSM | Courtemanche *et al.* (2019) | Evaluating the effects of Walmart Supercenters, which expand food availability at lower food prices, on food security | Program evaluation | Data from 396,000 households [Current Population Survey Food Security Supplement] | Linear probability models; instrumental variables estimation |
| HFSSM | Borch and Kjærnes (2016) ^‡^ | Assessing food insecurity prevalence and its determinants in in Nordic countries | Cause and effect analysis | Survey: 8,248 respondents from Norway, Denmark, Sweden, and Finland | Descriptive and linear regression analyses |
| HFSSM | Gregory and Coleman-Jensen (2013) | Investigating the effect of high food prices on household food security in the context of developed countries (US) | Cause–effect analysis | Panel data over 2002–2006 (observations: 177,434) [Current Population Survey food security Supplement and Quarterly Food-at-Home Price] | Endogenous switching regression model |
| HFSSM | Guo (2011) | Investigating the effects household assets on household food security status | Cause–effect analysis | Survey: 8,534 households in the US | OLS and logistic regression models |
| HFSSM | Bawadi *et al.* (2012) ^§^ | Assessing the prevalence and determinants of food insecurity among women in Jordan | Prevalence estimation, cause–effect analysis | Data collected from 500 women through interviews in two public hospitals | Descriptive analysis, multiple logistic regression model |
| ELCSA | Segall-Corrêa *et al.* (2018) ^¶^ | Developing an experience-based Brazilian food security scale for assessing food insecurity in indigenous communities | Prevalence estimation; cause–effect analysis | Survey: 88 indigenous Guarani households | Descriptive analysis; Rasch model |
| ELCSA | Romo-Aviles and Ortiz-Hernández (2018) | Assessing differences in food, energy and nutrients supplies among Mexican households according to their food insecurity status | Cause–effect analysis | Data from 19,479 households in Mexico [National Household Income and Expenditure Survey] | Ordinal regression and OLS models |
| ELCSA | Sandoval *et al.* (2020) | Comparing ELCSA and the household-undernourishment food security indicators in food security analyses | Prevalence estimation; cause–effect analysis; impact evaluation | Data from 13,531 households [Survey of Living Standards from Guatemala] | Logistic regression models |

^†^The 30-day version of HFSSM was used instead of the standard 12 months.

^‡^ An adapted HFSSM was used, where rather than using the 18-questions, only one question was used for measuring food security status, namely, ‘Which of these statements best describes the food eaten in your household in the last 12 months: [a] Enough of the kinds of food I/we want to eat, [b] Enough but not always the kinds of food I/we want, [c] Sometimes not enough to eat, [d] Often not enough to eat, and [e] DK or Refused’.

^§^ The authors used the short module (with six-items) of HFSSM.

^¶^ Segall-Corrêa *et al.* (2018) developed and validated a six-item questionnaire from the full version of ELCSA/Brazilian food security Scale for measuring food insecurity in Brazilian indigenous communities.

***Note***: Refer to the footnotes of Table S2 for the abbreviations.

**Table S5** Summary of the publications that used FIES

| Article | Objective of the study | Purpose of FIES use | Data | Method |
| --- | --- | --- | --- | --- |
| Tambo *et al.* (2021) | Effect of provision of plant health services on food insecurity prevalence | Program evaluation | Survey: 637 farm households (263 users and 374 non-clinic users) in Rwanda | Endogenous switching regression model; propensity score matching |
| Omidvar *et al.* (2019) | Assessing the prevalence and determinants of food insecurity in MENA countries | Prevalence estimation; cause–effect analysis | Data from 18,079 individuals [FAO’s FIES data from the 2016 wave of the Gallup World Poll] | Descriptive analysis; logistic regression models |
| Smith *et al.* (2017a) | Assessing the prevalence and determinants of worldwide food insecurity using FIES | Prevalence estimation; cause–effect analysis | Data from 123,732 individuals in 134 countries [FAO’s FIES data from the 2014 wave of the Gallup World Poll] | Multilevel/hierarchical linear probability models |
| Smith *et al.* (2017b) | Assessing the prevalence and determinants of food insecurity in Latin America and the Caribbean countries using FIES | Prevalence estimation; cause–effect analysis | Data from 17,188 individuals from the different countries [FAO’s FIES data from the 2014 wave of the Gallup World Poll] | Descriptive analysis; Multilevel/hierarchical linear models |
| Nicholson *et al.* (2021) | Analysing food security while considering all the four dimensions using agricultural systems models | Prevalence estimation; cause–effect analysis | Data on farm inputs, outputs, and household food access indicators and determinants (over time) | Dynamic farm household optimisation model |

***Note***: Refer to the footnotes of Table S2 for the abbreviations.
